# Utilizing Serum-Derived Lipidomics with Protein Biomarkers and Machine Learning for Early Detection of Ovarian Cancer in the Symptomatic Population

**DOI:** 10.1158/2767-9764.CRC-25-0140

**Published:** 2025-09-04

**Authors:** Brendan M. Giles, Rachel Culp-Hill, Robert A. Law, Charles M. Nichols, Mattie Goldberg, Enkhtuya Radnaa, Maria Wong, Connor Hansen, Moises Zapata, Collin Hill, Kian Behbakht, Benjamin G. Bitler, Emma J. Crosbie, Chloe E. Barr, Anna Jeter, Vuna S. Fa, Violeta Beleva Guthrie, Leonardo N. Hagmann, Emily C. Kubota, James Robert White, Abigail McElhinny

**Affiliations:** 1 AOA Dx, Denver, Colorado.; 2 University of Colorado Anschutz Medical Campus, Denver, Colorado.; 3 Division of Cancer Sciences, University of Manchester, Manchester, United Kingdom.; 4 Department of Obstetrics and Gynaecology, St Mary’s Hospital, Manchester University NHS Foundation Trust, Manchester Academic Health Science Centre, Manchester, United Kingdom.; 5 Resphera Biosciences, Baltimore, Maryland.

## Abstract

Ovarian cancer is the fifth leading cause of cancer-related deaths among women. Most patients are diagnosed at late stage (III/IV), resulting in a 5-year survival rate below 30%. This is driven by the presentation of vague abdominal symptoms that confound diagnosis at early stages (I/II) and a shortage of robust biomarkers. We are taking a novel approach for earlier ovarian cancer detection, leveraging lipids as biomarkers. We utilized untargeted ultrahigh pressure liquid chromatography–mass spectrometry to analyze sera from two large, independent cohorts (*N* = 433 and *N* = 399) designed to reflect the symptomatic population, including individuals with benign adnexal masses, early- and late-stage ovarian cancer, gastrointestinal disorders, and otherwise healthy women seeking care for symptoms. We identified a significantly altered lipid profile in ovarian cancer and early-stage ovarian cancer specifically across both cohorts compared with controls. We also profiled select protein biomarkers (cancer antigen 125, human epididymis protein 4, β-2 folate receptor α, and mucin 1) and, utilizing machine learning–based modeling, identified a proof-of-concept multiomic model consisting of less than 20 top-performing lipid and protein features. This model was trained on cohort 1 and tested on cohort 2, achieving AUCs of 92% (95% confidence interval, 87%–95%) for distinguishing ovarian cancer from controls and 88% (95% confidence interval, 83%–93%) for distinguishing early-stage ovarian cancer from controls. These findings demonstrate the clinical utility and robustness of lipids as proof-of-concept diagnostic biomarkers for early ovarian cancer within the clinically complex symptomatic population, particularly when applied in a multiomic approach.

**Significance:**

Patients with ovarian cancer endure delayed diagnosis and poor outcomes. We profiled lipids in two cohorts and integrated them with proteins in machine learning. This enabled early-stage detection in a complex range of controls.

## Introduction

Ovarian cancer is lethal, ranking as the fifth leading cause of cancer deaths in women (1, 2). Despite being 10 times less common than breast cancer, ovarian cancer is three times more deadly (3), largely because of late diagnosis (4, 5). It is also often mischaracterized as a “silent killer,” but a multitude of studies over the last decade have confirmed that more than 80% of individuals with early-stage (I/II) disease present with vague abdominal symptoms (VAS). These symptoms include abdominal and pelvic pain, common in a wide range of noncancerous conditions, including benign gynecologic conditions such as cysts, fibroids, and endometriosis. Many patients also undergo gastrointestinal (GI), general abdominal, and/or urological evaluations because ovarian cancer symptoms overlap with other non-gynecologic conditions (6). Vague symptoms coupled with a lack of effective diagnostic methods (Table 1) result in an average time to diagnosis in the United States of approximately 9 months (5–7). This diagnostic odyssey for patients with ovarian cancer frequently results in a series of clinical tests, including transvaginal ultrasound (TVU) and blood-based biomarker testing including cancer antigen 125 (CA125) levels, often repeated over several months of “watch and wait.” Meanwhile, fast-growing early-stage ovarian cancer tumors [including the most common form, high-grade serous (HGS)] can double in size every 4 months, and late-stage (III/IV) tumors can double in size every 2.5 months (8). This culminates in greater than 70% of patients diagnosed with ovarian cancer at late stages presenting with high tumor burden and a 5-year survival rate of 10% to 30% (9, 10). In stark contrast, if ovarian cancer is diagnosed earlier when tumors are localized, survival can jump to over 90% (6, 11). Recent evidence confirms that earlier diagnosis improves patient outcomes and survival rates (9, 12, 13). Consequently, strategies enabling earlier detection of ovarian cancer will result in considerable cost savings for the healthcare system, demonstrating clinical utility and improving patient outcomes (11).

**Table 1:** Patient journey

Method	Overall Sens. | Spec.	Description/application	Limitation
Ultrasound (TVU)	57% | 88% (53)	TVU imaging used to visualize pelvic organs Can detect masses in cervix, uterus, fallopian tubes, and ovaries	Small tumor size may not be detected until later stages Difficulty distinguishing benign vs. malignant masses with high accuracy Results vary by operator expertise (54)
CA125	79% | 78% (55)	Blood test to measure CA125 protein, commonly shed into the bloodstream by ovarian cancer cells Used as a tumor marker to detect ovarian cancer and monitor response to treatment (19)	Elevated levels associated with benign and other malignant conditions and limited sensitivity in early-stage ovarian cancer Levels may fluctuate because of age and noncancerous conditions (19) FDA-cleared for monitoring of disease after diagnosis only (56)
HE4	79% | 93% (55)	Blood test to measure HE4 protein secreted by epithelial ovarian cancer cells Used as a tumor marker to detect ovarian cancer and monitor response to treatment (57)	Elevated levels associated with benign and malignant conditions and limited sensitivity in early-stage ovarian cancer (57) Levels vary by smoker status and hormonal contraceptive use (58) FDA-cleared for monitoring of disease after diagnosis only, limited availability (56)
OVA1	92% | 50% (59)	Blood test to measure CA125 + four biomarkers, integrates clinical information into algorithm Distinguishes benign vs. malignant masses in individuals scheduled for surgery (59)	Reduced sensitivity in premenopausal individuals with low-risk CA125, modest overall specificity, and high false-positive rate (59) Dependency on menopausal state FDA-cleared for triaging adnexal mass already scheduled for surgery (60)
Overa	91% | 66% (59)	Blood test to measure CA125 + four biomarkers, integrates clinical information into algorithm, and reflex test for OVA1 Distinguishes benign vs. malignant masses in individuals scheduled for surgery (59)	Reflex test to OVA1 Modest overall specificity and high false-positive rate Reduced specificity for post-menopausal individuals (59) FDA-cleared for triaging adnexal mass already scheduled for surgery (60)
ROMA	74% | 93% (59)	Blood test to measure CA125 + HE4 protein levels, integrates menopausal status into algorithm Classifies patients as high or low risk Distinguishes benign vs. malignant masses in individuals with ovarian adnexal mass (19)	Moderate overall sensitivity Reduced sensitivity in pre-menopausal individuals (59) Reduced sensitivity for early-stage ovarian cancer FDA-cleared for triaging adnexal mass already scheduled for surgery (60)

Overview of the current standard of care and diagnostic testing representative of the journey of a patient with ovarian cancer.

Given the relatively poor sensitivity and specificity of current ovarian cancer diagnostic methods, novel approaches are urgently needed to improve the clinical workflow for diagnosing early-stage ovarian cancer. We previously identified tumor marker gangliosides as potential novel biomarkers for ovarian cancer (14). Gangliosides are a subclass of sphingolipids, broadly expressed on cell surfaces and shed into circulation (14, 15). Our initial work focused on two gangliosides, GD2 and GD3, which exhibited promising results in distinguishing ovarian cancer from non–ovarian cancer specimens in a serum-based immunoassay platform (15). However, as signs and symptoms of ovarian cancer are shared across a broad spectrum of non–ovarian cancer conditions, we hypothesized that the complexity of disentangling molecular signals from early-stage ovarian cancer in this heterogeneous population would require a combination of multiple biomarker classes. In this regard, multiomic approaches paired with machine learning (ML) modeling are promising as proof of concept in orthogonally detecting cancer signals generated from various molecular processes that would be missed by single biomarkers alone (16). As other lipid classes beyond gangliosides demonstrate specific alterations in ovarian cancer (17), we aimed to take a broad-spectrum approach to identify as many putative lipid biomarkers as possible and evaluate their potential complementary clinical performance.

Recent studies characterizing lipid alterations in ovarian cancer show the potential of lipids as a novel class of biomarkers that moves past the longstanding standard of care (SOC), CA125. In fact, applying a combinatorial approach of known protein biomarkers with analytes such as plasma lipids (18) or additional proteins (19) improves diagnostic performance for ovarian cancer. We hypothesized that using ML to combine untargeted lipidomics with a few protein markers would enhance performance in the complex VAS population in a proof-of-concept study. This approach would represent a significant advancement in identifying early-stage ovarian cancer in individuals presenting with symptoms, addressing a critical unmet clinical need.

## Materials and Methods

### Cohort design

The base population of women experiencing VAS in the United States reflects a broad spectrum of clinical presentations, including noncancerous gynecologic conditions and various GI disorders for which symptoms can overlap with the ovarian cancer symptom index (20). With the goal of identifying novel biomarkers that can distinguish ovarian cancer within this complex population (21, 22), we designed two independent cohorts enriched with ovarian cancer serum specimens across stages and subtypes. We obtained clinically annotated frozen serum samples from various sources and included ovarian cancer, benign (noncancerous) gynecologic conditions such as endometriosis, fibroids, cysts, and other adnexal masses (23), and GI conditions that included gastritis, colitis, and Crohn disease. Age-representative, otherwise healthy female donors were included to compare baseline lipid and protein levels. Representative noncancer conditions from these cohorts were grouped into “all controls,” and samples across stages and subtypes of ovarian cancer were grouped into “all cancer,” which was further delineated by early-stage (I/II) and late-stage (III/IV) as described in Supplementary Table S1 (see Supplementary Table S2 for cohort clinical and demographic details).

Cohort 1 samples were obtained from the University of Colorado Gynecologic Tissue and Fluid Bank (Institutional Review Board #07-935 and 21-4787) and a commercial vendor. Cohort 2 samples included a biobanked subset of the University of Manchester England DETECT study (IRAS ID 208785; ref. 24), collected prospectively from women seeking care for a wide range of symptoms. All participants gave written informed consent in accordance with the Declaration of Helsinki, and all samples were de-identified before being provided for this study. Prospective collection refers to obtaining samples before surgical intervention or treatment, with participants enrolled and biospecimens collected prior to clinical outcome determination. Cohort 2 was also supplemented with samples from commercial biobanks.

Samples from vendors were selected based on predefined criteria to mitigate sample quality variability, where possible. Serum samples were excluded based on the following criteria: subjected to >2 freeze–thaw cycles, obvious hemolysis or particulates, individuals with a current cancer diagnosis other than ovarian cancer, individuals with a previous ovarian cancer diagnosis, and individuals who were pregnant at the time of collection. Although selections obtained from commercial sources represent biological females diagnosed with conditions described in Supplementary Table S1 and who presented with VAS aligning with the ovarian cancer symptom index, samples were not prospectively collected based on this index. All selected individuals were not undergoing cancer-related treatment when blood was obtained for these specimens. For each sample, available data included cancer diagnosis, stage, and subtype (for ovarian cancer cases), as well as age, sex, ethnicity, date of diagnosis, comorbidities, smoking history, and if available, medical history, presented symptoms, and symptom duration.

### Sample extraction

For lipid profiling, serum was extracted using 100% LC/MS-grade methanol (Thermo Fisher Scientific). This method was determined to yield reproducible lipid recovery across operators and days. Methanol was spiked with GD1b Ceramide (d18:1/18:0-d7), GD3 Ceramide (d18:1/18:0-d7), 15:0/18:1-d7 PE, 18:1-d7 LysoPE, and 18:1-d9 SM.

Into 2 mL Eppendorf tubes, 20 μL serum was aliquoted. To each tube, 180 μL of methanol solution spiked at room temperature was added. Samples were vortexed at room temperature at maximum speed for 30 seconds and then centrifuged at 18,000 rcf for 10 minutes at 4°C. The supernatant was transferred to autosampler vials containing 300 μL glass inserts. From every fifth sample, 5 μL supernatant was removed in randomized order and added to a technical pooled mixture for global sample-level monitoring. Samples were stored at −20°C until LC/MS analysis.

### LC/MS specifications

Commercial reagents were purchased from Thermo Fisher Scientific. Analysis was performed by ultrahigh pressure liquid chromatography–mass spectrometry (using Vanquish (RRID: SCR_025713) and Exploris 240 (RRID: SCR_022216) systems, Thermo Fisher Scientific) using C18 reversed-phase chromatography and electrospray ionization. A total of 15 μL sample extract was loaded onto a Kinetex 2.6 μm C18 100Å LC Column (100 × 2.1 mm – Phenomenex) at 40°C. A 20-minute gradient at 320 μL/minute (40%–90% B, A: 60:40 methanol/water + 10 mmol/L ammonium formate, B: 90:10 isopropanol/methanol + 10 mmol/L ammonium formate) was used to elute lipids of interest. For negative mode, three separate mass spectrometry (MS) experiments were run within the gradient.

1. 0 to 4 minutes: full scan at 180,000 resolution with scan range from 90 to 500 m/z.
2. 0 to 4 minutes: full scan at 120,000 resolution with scan range from 500 to 1,700 m/z.
3. 4 to 20 minutes: full scan at 120,000 resolution with scan range from 500 to 1,700 m/z.

Data-dependent MS2 was performed using five dependent scans at 22,500 resolution with a scan range from 90 to 900 m/z (experiments 2 and 3). Higher-energy collisional dissociation collision energy (%) was set to 20 with a 2 m/z isolation window.

For positive mode, full scan was performed at 120,000 resolution with a 300 to 1,700 m/z scan range. Data-dependent MS2 was performed using an 800 ms cycle time with 30,000 resolution and automatically detected scan range. Higher-energy collisional dissociation collision energy (%) was set to 20 and 30 with a 1.5 m/z isolation window. EASY-IC was used for full scan experiments in both modes. Calibration was performed before analysis using Pierce FlexMix Calibration Solution (Thermo Fisher Scientific). A technical pooled mixture was injected every 10 samples to assess LC/MS precision. Internal standards maintained a coefficient of variation (CV) below 10% throughout the run.

### LC/MS data analysis

For ganglioside and fatty acid analysis, data files (.raw) were converted to .mzML format using MS Convert (ProteoWizard, v3.0). Assignments were performed using isotopologue distributions and expected natural abundances of ^13^C isotopes using MAVEN (v2.10.21, RRID: SCR_022491). Ganglioside identifications were confirmed using ganglioside-specific fragment moieties. CompoundDiscoverer (v3.3 SP3, Thermo Fisher Scientific) was used for relative quantitation and lipid assignment against publicly available lipid databases. Databases used for putative library matches included LipidMaps, HMDB, mzCloud, mzVault, MassList, and ChemSpider. Feature spaces underwent filtering steps which included exclusion of (i) features without a putative ID through library matching, (ii) features present in less than 10% of samples within a cohort, (iii) features of exogenous origin, including drugs, synthetic compounds, dietary metabolites, and non-microbiome bacterial products, and (iv) features exhibiting high technical variability (CV > 50%) across the technical quality control sample (Supplementary Fig. S1, steps 1–4.). In cohorts 1 and 2, 2,795 and 2,015 compounds were retained, respectively, prior to lipidomic analysis (see “Lipidomic Profiling”) or further feature selection for statistical modeling (see “Feature Reduction, Normalization, and Statistical Modeling”). Partial least squares discriminant analysis (PLSDA) score plots and heatmaps were generated using Metaboanalyst (RRID: SCR_015539; ref. 25). Scatter plots and statistical analyses were performed in Prism (GraphPad v10.4, RRID: SCR_002798). Statistical testing used Mann–Whitney U with significance defined as ns (*P* > 0.05); ** (*P* ≤ 0.01); *** (*P* ≤ 0.001); **** (*P* ≤ 0.0001).

### Protein immunoassays

Commercially available immunoassay kits were obtained from R&D Biosystems (Bio-Techne) for CA125, human epididymis protein 4 (HE4), and β-2 folate receptor α (FOLR1) and from Thermo Fisher Scientific (Invitrogen) for mucin 1 (MUC1). Each kit was analytically verified prior to use per manufacturer’s instructions. Each immunoassay was performed on unextracted serum from each individual specimen. Repeat testing was performed as needed for high variability replicates (>20% CV). Scatter plots and statistical analyses were performed in Prism (GraphPad v10.4, RRID: SCR_002798). Statistical testing used Mann–Whitney U with significance defined as ns (*P* > 0.05), ** (*P* ≤ 0.01), *** (*P* ≤ 0.001), **** (*P* ≤ 0.0001).

### Sample exclusions

Although initial sample selection included 487 samples in cohort 1 and 402 samples in cohort 2, sample attrition occurred during the analytic workflow because of technical issues with assignable cause that prevented or disrupted data acquisition. These issues included sample volume extinction, out of specification internal control results, instrument errors, and corrupt data files.

For cohort 1, 54 samples were excluded: nine for sample depletion during LC/MS, one for a corrupted LC/MS data file, and 44 because of increased instrument signal variability. For cohort 2, three samples were excluded because of an instrument processing error. Taken together, 54 of the 487 samples originally allocated to cohort 1 were excluded, resulting in 433 samples with complete datasets eligible for modeling. For cohort 2, three of the 402 samples originally allocated were excluded, resulting in 399 samples with complete datasets eligible for modeling. Total analyzed and allocated samples are shown in Table 2.

**Table 2:** Cohort sample distribution before and after sample exclusions

Diagnosis	Group	Cohort 1	Cohort 2	Combined
Cancer	All ovarian cancer	159/187	108/109	267/296
	Early-stage ovarian cancer	60/74	52/52	112/126
	Late-stage ovarian cancer	99/113	56/57	155/170
Noncancer/controls	All controls	274/300	291/293	565/593
	Normal	75/82	206/208	281/290
	Benign conditions	155/168	85/85	240/253
	GI disorders	44/50	0/0	44/50
Grand totals:		433/487	399/402	832/889

Summary table of ovarian cancer and non–ovarian cancer conditions included to represent the symptomatic population. Totals are displayed as the number analyzed over the number allocated, as described in Sample Exclusions.

### Feature reduction, normalization, and statistical modeling

The total number of identified lipid analytes (2,795 and 2,015 in cohorts 1 and 2, respectively) was systematically filtered as described in “LC/MS Data Analysis” and illustrated in Supplementary Fig. S1. Feature spaces underwent filtering steps which included exclusion of (i) features without a putative ID through library matching, (ii) features present in less than 10% of samples within a cohort, (iii) features of exogenous origin, including drugs, synthetic compounds, dietary metabolites, and non-microbiome bacterial products, and (iv) features exhibiting high technical variability (CV >50%) across the technical quality sample sample (Supplementary Fig. S1, steps 1–4). To identify features with potential to function as reproducible clinical biomarkers for ovarian cancer, the features were further reduced to include only those detected in both cohorts (step 5). Therefore, the final number of lipid features available for statistical modeling was 973. For analytes that passed the initial filtering steps, any missing values in individual samples were imputed by adding a constant equal to 10% of the lowest detected value of that analyte. Analyte abundance levels were then log transformed.

In statistical modeling using untargeted LC/MS acquired across multiple cohorts and time points without internal standards for identification and quantitation, normalization is critical to correct for batch effects and analytic drift. Variability is introduced through changes in instrument performance, ion suppression, column performance, and sample preparation, which can confound biological interpretations in multi-cohort studies. Normalization allows for pooling datasets collected at different times, allowing for increased scale of LC/MS-based lipidomics experiments and mitigating misinterpretation of results (26). Additionally, because of the scale of features detected with untargeted analysis, it is not feasible to obtain standards for each metabolite for individual feature normalization. Therefore, batch effects between training (cohort 1) and testing (cohort 2) cohorts were mitigated using a two-step normalization approach: batch correction followed by signal standardization.

First, for batch correction, a modified ComBat (RRID: SCR_010974) harmonization approach was applied to each LC/MS analyte separately to correct for batch-related location and scale differences (27). Correction parameters including global location (mean shift) and scale correction (variance normalization) parameters, along with their empirical priors, were computed using a set of 50 samples included with both cohorts (Supplementary Table S3). The 50 samples from cohort 1 were used as part of feature selection and training in cohort 1. They were then re-extracted and processed with cohort 2 and used for normalization purposes only. These parameters were then applied to the full cohort dataset, with cohort 1 serving as the reference cohort. Next, signal standardization using sample level *z*-score scaling was applied to standardize intensity distribution within each sample. Protein immunoassay analytes were not batch corrected or *z*-scored as they did not exhibit cohort-level batch effects requiring normalization.

Features were ranked according to discriminant power using area under the ROC curve (AUC) achieved by distribution between cancer versus benign and cancer versus normal conditions in the training cohort. The top 200 ranked features were selected for initial evaluation in the training set based on univariate discriminate power in cohort 1. An elastic net regularized logistic regression model (α = 0.9) was fitted to the training cohort using 20-fold cross-validation with five repeats. The top 10% features with the highest average importance from this model were selected for final evaluation. The elastic net model (α = 0.9) fit to the training cohort (cohort 1) was evaluated on the testing cohort (cohort 2). Features with non-zero coefficients after regularization represent the final feature set. Analyses were performed using R v4.4.2 (28).

The above analysis was executed with multiple configurations of key hyperparameters in the modeling procedures, for example, leaving out sample normalization or batch correction, using standard logistic regression instead of elastic net or selecting a different proportion of features during model refinement. Thus, the selected model represents a slightly optimistic estimate of generalized performance; however, performance across other parameter combinations was not dramatically inferior.

### Data availability

All data are available in the main article, supplemental files, or upon request from the corresponding author.

### Ethics

Informed consent for all samples was obtained by external institutions and vendors. For external institutions’ protocol, refer to the University of Colorado Gynecologic Tissue and Fluid Bank (Institutional Review Board #07-935 and 21-4787) and the University of Manchester England DETECT study (IRAS ID 208785).

## Results

### Lipidomic profiling

Lipidomics has become increasingly relevant since aberrant lipid metabolism was identified as a cancer hallmark, with various disease states displaying unique lipid profiles (17, 18, 29). As our initial work focused on tumor marker gangliosides, we first analyzed serum samples from both cohorts for alterations in the ganglioside profile, also known as the “gangliosome” (14, 15). Comparing ganglioside profiles between all ovarian cancer stages and subtypes (“ovarian cancer”) and the wide range of clinical non–ovarian cancer controls (“controls”), we observed alterations in the ganglioside profile when comparing ovarian cancer with controls when looking at the top 50 by ANOVA. We also replicated previously observed shifts in levels of GD2 and GD3, but these shifts were subtle in both cohorts, indicating that a wider range of lipids are required to distinguish ovarian cancer in the symptomatic population (Supplementary Figs. S2 and S3). To cast a wide net for biomarker identification, our untargeted LC/MS method was also designed to detect a broad range of lipids (Supplementary Fig. S4). An untargeted approach avoids limitations inherent to a targeted method, in which only known analytes can be measured. We increased confidence in identifying putative lipid features that are biologically relevant biomarkers by ensuring they were present in each dataset.

**Figure 1:**
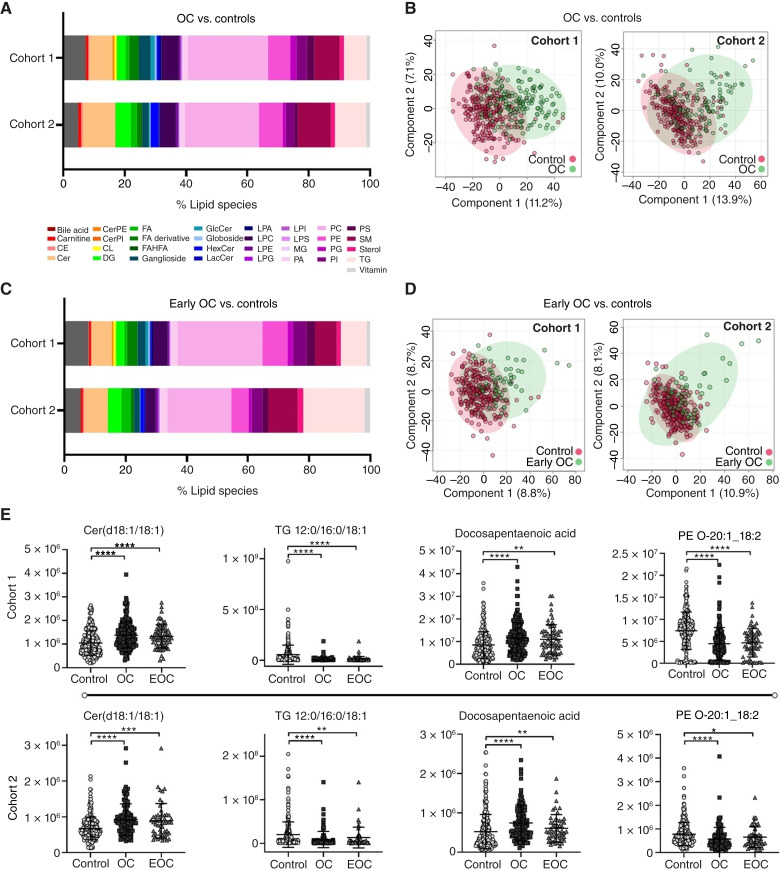
Lipidomics analysis. **A,** Lipid class assignments as a percentage of species reaching *P* < 0.05 by the Mann–Whitney u test comparing controls with ovarian cancer. **B,** PLSDA analysis of controls and ovarian cancer (OC) for each cohort. **C,** Lipid class assignments as a percentage of species reaching *P* < 0.05 by the Mann Whitney U test comparing controls with early-stage ovarian cancer. **D,** PLSDA analysis of controls and early-stage ovarian cancer (Early OC) for each cohort. **E,** Scatter plots depicting relative abundance of representative lipid species across cohorts 1 and 2 for control, ovarian cancer (OC), and early-stage ovarian cancer (EOC). Cer, ceramide; CL, cardiolipin; DG, diacylglycerol; FFA, free fatty acid; GlcCer, glucosylceramide; GM3, GM3 ganglioside; LacCer, lactosylceramide; LPA, lysophosphatidic acid; LPC, lysophosphatidylcholine; LPE, lysophosphatidylethanolamine; LPG, lysophosphatidylglycerol; LPI, lysophosphatidylinositol; LPS, lysophosphatidylserine; MG, monoacylglycerol; PA, phosphatidic acid; PC, phosphatidylcholine; PE, phosphatidylethanolamine; PG, phosphatidylglycerol; PGP, phosphatidylglycerophosphate; PI, phosphatidylinositol; PS, phosphatidylserine; SM, sphingomyelin; TG, triacylglycerol. Statistical testing used Mann Whitney U with significance defined as: ns, *P* > 0.05; **, *P* ≤ 0.01; ***, *P* ≤ 0.001; ****, *P* ≤ 0.0001.

As an exploratory interrogation of lipid profiles between ovarian cancer and controls, we filtered the dataset to include statistically significant species between groups for each cohort individually (Fig. 1A). A total of 1,845 and 1,050 lipids were significantly altered in ovarian cancer versus controls from cohorts 1 and 2, respectively, with a similar pattern of altered lipid classes observed across both cohorts. Heatmaps show distinct clustering patterns, indicating significant differences in the ovarian cancer serum lipid profile (Supplementary Fig. S5). PLSDA further demonstrated separation between ovarian cancer and controls (Fig. 1B), with score plots showing clear separation along the first two component variables. Corresponding variable importance in projection scores (Supplementary Fig. S6) highlight key lipid features driving separation and include phospholipids, sphingomyelins, and ceramides. These data indicate that there are significantly altered lipid classes across stages and subtypes of sera from patients with ovarian cancer, even when profiled against the complex, wide range of individuals experiencing signs and symptoms of ovarian cancer.

As lipidomic profiles shift with progression of disease (18), we interrogated the profile of early-stage ovarian cancer serum specifically. Data were filtered to include statistically significant species between early-stage ovarian cancer and controls. (Fig. 1C) In cohorts 1 and 2, 1,262 and 455 lipids were significantly altered, respectively, and we again observed a similar pattern of altered lipid classes between cohorts. Heatmaps and PLSDA show less distinct clustering patterns between early-stage ovarian cancer and controls (Fig. 1D). However, corresponding variable importance in projection scores (Supplementary Fig. S6) show that although individual lipid species differ between ovarian cancer and early-stage ovarian cancer, the lipid classes driving separation (e.g., phospholipids, sphingomyelins, and ceramides) are common across both comparisons. Additionally, heatmaps and PLSDA for controls, early-stage ovarian cancer, and late-stage ovarian cancer show a clear shift by stage, underscoring the progressive changes in the lipidomic profile associated with advancement of ovarian cancer (Supplementary Fig. S7).

As discussed, cohorts 1 and 2 are clinically and demographically distinct, particularly in control heterogeneity. However, despite these biological differences, ovarian cancer and early-stage ovarian cancer retain separation from controls, suggesting that early-stage ovarian cancer serum can be biochemically differentiated from control serum. Although this divergence is least visible in cohort 2, it is clearly observed for individual lipid species (Fig. 1E). For example, in ovarian cancer and early-stage ovarian cancer, triacylglycerol (12:0/16:0/18:1) and phosphatidylethanolamine (O-20:1/18:2) are significantly decreased whereas ceramide (d18:1/18:1) and docosapentaenoic acid are significantly increased compared with controls.

The significant differences in individual lipid features in both ovarian cancer and early-stage ovarian cancer serum compared with controls across cohorts suggest that individual features can be exploited to distinguish control and ovarian cancer serum. Considering the subtle global lipidomic differences observed between controls and early-stage ovarian cancer, especially in cohort 2, individual biomarker classes such as lipids could benefit from orthogonal signals for robust early-stage ovarian cancer detection. We therefore hypothesized that a clinical diagnostic assay to detect ovarian cancer in the symptomatic population would benefit from inclusion of protein biomarkers in addition to untargeted lipids.

### Protein biomarker levels

Four protein biomarker targets were identified for testing in both cohorts: CA125, HE4, FOLR1, and MUC1. CA125 and HE4 are currently used in clinical practice (Table 1), whereas FOLR1 and MUC1 show promise as diagnostic and therapeutic targets for ovarian cancer (30–32). The selected markers were assessed in both cohorts with protein-based immunoassays that were analytically verified to meet standard analytic performance metrics.

**Figure 2:**
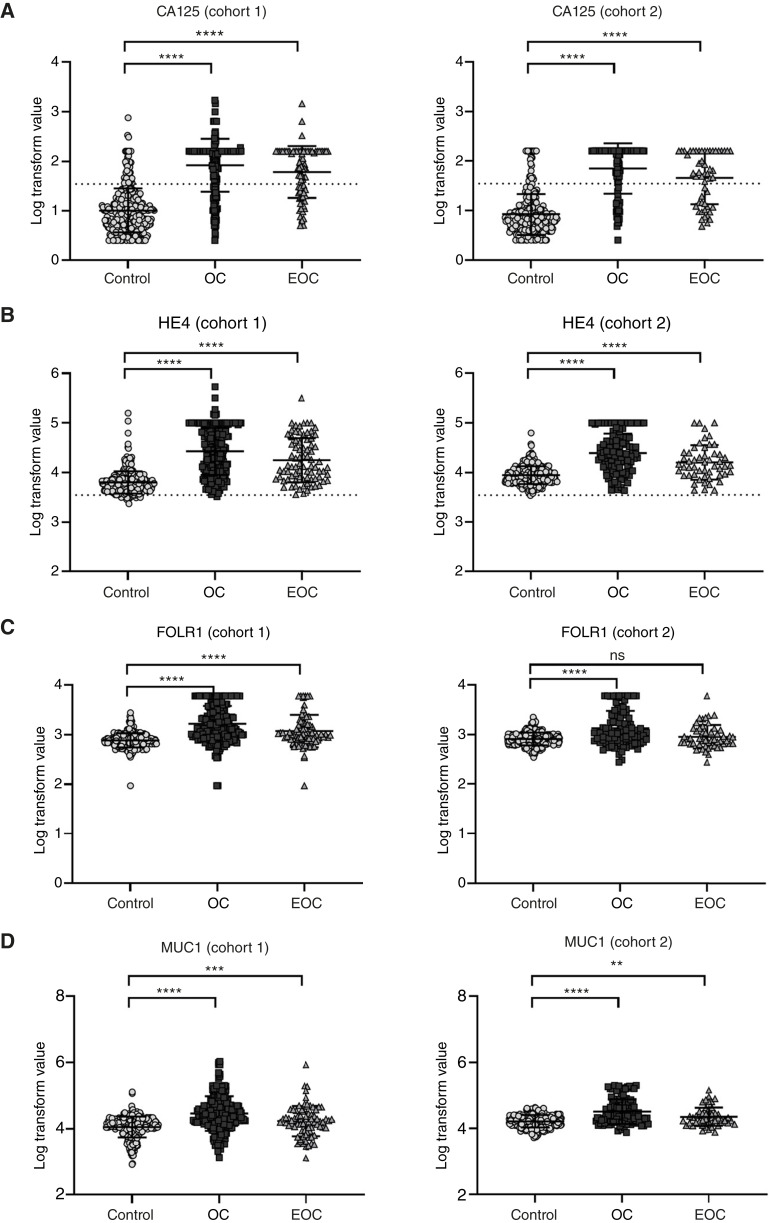
Protein biomarker levels. Protein biomarkers were quantified via immunoassay, and values are log_10_ transformed and visualized by scatter plot. **A,** CA125. Dotted line shows threshold referenced in clinical guidance (35 U/mL). **B,** HE4. Dotted line shows threshold referenced in clinical guidance (140 pmol/L). **C,** FOLR1. **D,** MUC1. Error bars indicate mean and SD. Mann–Whitney U tests were performed between controls and each ovarian cancer group. OC, ovarian cancer. EOC, early-stage ovarian cancer. Statistical testing used Mann Whitney U with significance defined as: ns, *P* > 0.05; **, *P* ≤ 0.01; ***, *P* ≤ 0.001; ****, *P* ≤ 0.0001.

For cohort 1, all protein biomarkers were increased in both ovarian cancer and early-stage ovarian cancer as compared with controls (Fig. 2). Protein biomarker performance in cohort 2 was similar, with increases observed for all markers when comparing ovarian cancer and controls (Fig. 2). For cohort 2, CA125, HE4, and MUC1 were increased in early-stage ovarian cancer whereas FOLR1 was equivalent between early-stage ovarian cancer and controls (Fig. 2). Overall, protein biomarker performance was similar in both cohorts: established markers CA125 and HE4 showed expected increases for cancer groups regardless of stage, with CA125 having the largest effect size. Exploratory markers FOLR1 and MUC1 had subtle increases for cancer groups, with early-stage ovarian cancer samples having the smallest effects.

Wide ranges and modest differences in mean levels highlight the limitations of relying on any of these proteins, including CA125 and HE4, as standalone targets for ovarian cancer. Although group-level differences are statistically significant, substantial overlap between cases and controls limits clinical utility when applying standard thresholds. Consistent with this, the application of current clinical guidelines for CA125 to both cohorts results in large numbers of controls falling above the clinical threshold and both ovarian cancer and early-stage ovarian cancer samples falling below the clinical threshold (Fig. 2). These findings underscore the limitations of protein biomarkers alone, including traditional diagnostic targets, and suggest complementary orthogonal biomarkers such as lipids are necessary to improve diagnostic performance.

### Feature space filtering

Although lipidomic profiling and protein biomarkers show univariate differences between cancer and control groups, wide distributions and subtle changes, particularly in early-stage ovarian cancer, illustrate the need for combinatorial approaches for modeling. To enable ML modeling, the feature space underwent filtering steps as described in “LC/MS Data Analysis” (Supplementary Fig. S1). The space was narrowed through defined criteria for lipid profiling described above to 2,795 features in cohort 1 and 2,015 features in cohort 2. To further down-select features to those with potential to function as potential clinically relevant biomarkers, we next determined which were enriched (overlapping) in both cohorts. Although not standard practice for targeted assays, requiring analytes to be detected in both cohorts helps mitigate the inherent variability and semi-quantitative nature of untargeted LC/MS, thereby increasing confidence in consistent feature identification and strengthening the reliability of putative identifications. By focusing on features detected in both cohorts, the approach taken here leverages the full potential of a second untargeted analysis to prioritize consistently observed signals rather than limiting discovery to features observed only once. The large number of nonoverlapping features (2,864) between the two cohorts is unsurprising based on the known demographic, clinical, and source differences (see Supplementary Table S2). Filtering to include only overlapping compounds therefore enriches for reproducible biomarkers across heterogeneous populations and enables untargeted datasets to be mined more efficiently to identify the best candidates for further development. The 973 common compounds comprise 865 lipids, 18 fatty acids, and 90 gangliosides, representing the lipid input for ML model training. The initial feature space contained 973 lipids and four protein biomarkers, totaling 977 features. This list was further narrowed by selecting the top 200 features based on univariate discriminate power in cohort 1 as described.

### Multiomic model performance

Although lipidomic profiling and protein biomarkers show univariate differences between cancer and control groups, wide distributions and subtle changes, particularly in early-stage ovarian cancer, illustrate the need for combinatorial approaches for modeling. ML-based modeling was therefore performed to assess the effectiveness of a multiomic approach for distinguishing ovarian cancer from the wide range of VAS controls.

**Figure 3:**
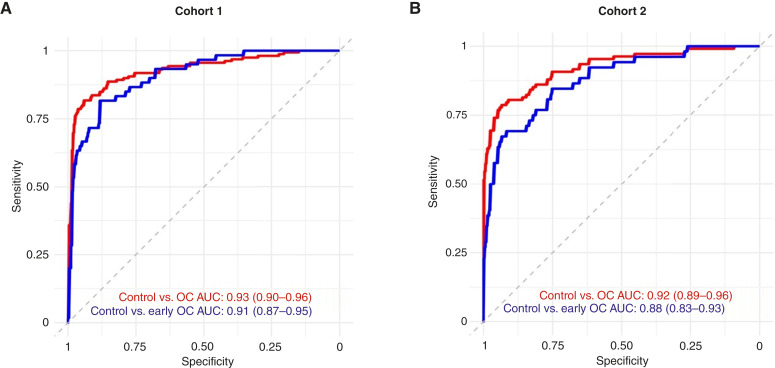
Multiomic model performance. ROC curves for distinguishing cancer from noncancer in cohort 1 (training; **A**) and cohort 2 (test; **B**). The AUC is shown for each comparison: control vs. ovarian cancer (red) and control vs. early-stage ovarian cancer (blue). OC, ovarian cancer.

Cohorts were used as distinct sets, with cohort 1 used for model training and cohort 2 used for model testing. To address the inherent variability of untargeted lipidomics, a two-step normalization process was deployed. Briefly, 50 common samples from cohort 1 were independently re-extracted and re-analyzed alongside cohort 2 samples. These common samples enabled batch correction without compromising the analytic independence of cohort 2. The 50 common samples were utilized solely for normalization between cohorts and were not utilized in modeling for cohort 2. *Z*-score signal standardization was then performed on feature intensity to standardize within each sample. ROC curves were generated using normalized data, and AUC was determined to assess model performance in terms of accuracy in distinguishing cancer from controls for both ovarian cancer and early-stage ovarian cancer groups (Fig. 3). Of the top 200, less than 20 features (a mix of lipids and proteins) were identified as “high importance” and used in downstream analysis (see “Statistical Modeling”)*.* For cohort 1, the top-performing model (with 20-fold cross-validation) resulted in AUCs of 93% for controls versus ovarian cancer and 91% for controls versus early-stage ovarian cancer (Fig. 3A). This underscores the enhanced discriminatory power gained by integrating multiomic biomarker data, especially compared with previously observed performance for CA125 alone of just 76% in distinguishing early-stage ovarian cancer from benign tumors (33). Despite differences in clinical characteristics and demographics between cohorts, the model maintained high accuracy when tested on cohort 2 as a hold-out set, achieving AUCs of 92% for controls versus ovarian cancer and 88% for controls versus early-stage ovarian cancer (Fig. 3B).

The robust model performance when using heterogeneous cohorts as discrete training and test sets confirms prior independent observations that lipid profiles and individual proteins are altered between ovarian cancer and control groups and suggests that subtle individual differences can be combined to enhance cancer detection. Furthermore, the observation that a small number (less than 20) of lipid and protein analytes can perform consistently across a wide range of clinical presentations highlights the potential real-world clinical application of this approach. Evaluations of lipid-only and protein-only models demonstrated lower performance than the proof-of-concept multiomic model for distinguishing early-stage ovarian cancer from controls (AUCs were consistently below 0.90 for single omics). Taken together, these data illustrate the potential power of a multiomic approach that leverages lipid profiling with just a few proteins to distinguish ovarian cancer in complex cohorts.

## Discussion

Ovarian cancer is often mischaracterized as a “silent killer.” In reality, patients present with VAS at early stages and spend ∼9 months navigating the healthcare system before receiving a diagnosis, leading to high mortality and poor prognosis (6, 11). This demonstrates a clear and critical unmet need for early detection diagnostic tools to change the ovarian cancer landscape and improve patient outcomes. Our approach aims to address a critical gap in clinical practice and improve the rate of successful intervention. Recent evidence demonstrates that testing for ovarian cancer in symptomatic individuals achieves early detection rates comparable with costly screening programs without population-wide initiatives (13). In particular, a fast-track pathway for symptomatic individuals led to early-stage (I/II) diagnoses in 25% of HGS ovarian cancer cases, a subtype traditionally associated with late-stage presentation and poor prognosis (13). Importantly, the majority of patients diagnosed with early-stage ovarian cancer through this fast-track pathway maintained good (0 or 1) performance status and achieved favorable surgical outcomes, with complete cytoreduction or minimal residual disease in more than 75% of cases (13). A more sensitive test would significantly affect overall outcomes by further improving the rate of early-stage diagnosis, reducing diagnostic time, and ensuring that surgery is performed by an appropriate provider. These findings challenge the assumption that symptomatic presentation indicates advanced disease, highlighting the critical role of accurate and sensitive diagnostic tools in expediting patient evaluation. Early intervention in this population is not only feasible but essential for improving survival outcomes. A study of nine European healthcare systems indicates that a 30-day reduction in time to diagnosis is associated with a 5% increase in the 5-year survival rate (6). Similarly, a study on diagnostic timelines showed a 17% to 18% decrease in 5-year survival due to a 3-month delay driven by the impact of the COVID-19 pandemic (12). For individuals presenting with signs and symptoms of ovarian cancer, introducing a diagnostic test with greater sensitivity and specificity than the current SOC has significant potential to drive earlier detection, reduce diagnostic delays, and generate economic benefit to the healthcare system.

Current SOC diagnostic tools consist of a CA125 blood test combined with imaging modalities such as TVU (19, 34). However, each method has limitations and neither is reliable for early detection. CA125 is elevated in noncancerous conditions such as endometriosis, fibroids, liver disease, and menstruation, leading to fluctuations, false positives, patient anxiety, and unnecessary interventions (19, 35–38). Although TVU is useful for detecting large ovarian tumors (39), it cannot reliably detect ovarian cancer at early stages, cannot distinguish malignant from benign tumors (40), and is limited by operator skill and subjectivity (39, 41). A definitive diagnosis of ovarian cancer requires surgery and removal of the ovary and fallopian tube with biopsy of the tumor; therefore, better triage tools earlier in the patient journey are essential. Although advances have been preliminarily reported for ovarian cancer detection in asymptomatic individuals (42, 43), the biologically complex symptomatic population requires novel approaches. Multiomics has the potential to identify early-stage disease using orthogonal molecular signatures that could be missed when analyzing individual biomarker classes alone (44). A robust, non-invasive, early detection diagnostic test for the symptomatic population would significantly improve the current poor prognosis of ovarian cancer.

Our previous work has shown that two novel tumor marker gangliosides, GD2 and GD3, are markers for detection of ovarian cancer for all stages and subtypes, including early stages I and II and HGS (15). However, other lipids are deeply involved in cancer pathways: lipid reprogramming is a known hallmark of cancer (45), lipids influence tumor suppression (46), and PI3K mutations affecting the PI3K/AKT/mTOR pathway, responsible for cell division and growth; ref. 47) result in alterations in several lipid classes (48). Recent breakthroughs in MS are also enabling deep lipidomic profiling studies. For example, sub-1 parts per million mass accuracy is improving resolution of isomeric lipid species in human serum and tumor microenvironments and improved sensitivity allows for detection of low-abundance lipids, novel high-throughput workflows are supporting efficient analysis of large clinical cohorts, and expanding databases are supporting more confident lipid annotation. These advancements have aided exploration of previously inaccessible parts of the lipidome, revealing novel biomarkers hidden within subtle lipidomic alterations. Lipids can serve as therapeutic or diagnostic targets, ushering in a new era of molecular profile–based diagnostics. In line with these developments, untargeted MS-based lipidomics has recently been harnessed to profile patients with pancreatic cancer within a three-phase study across three independent sites (29) and a recent review shows the clinical potential of lipids for ovarian cancer diagnostic applications (17).

**Figure 4:**
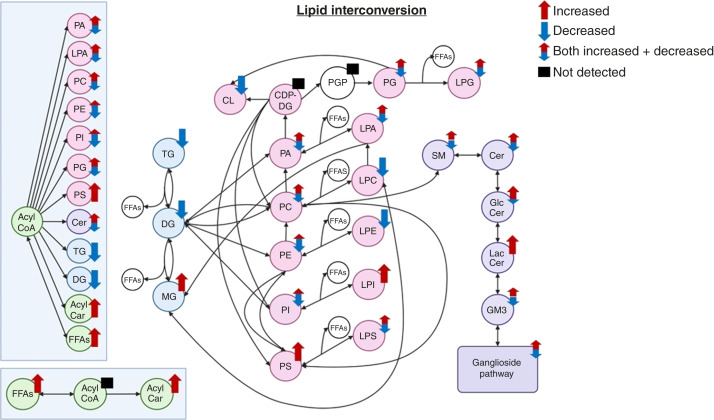
Metabolic interconversion of lipid species in human serum. Metabolic map illustrating the biochemical pathways governing the interconversion of major lipid species. Lipid metabolism is interconnected through pathways such as glycerophospholipid metabolism, sphingolipid metabolism, and fatty acid β-oxidation. Lipid classes are color-coded based on their biochemical category to facilitate visualization of metabolic flux. Across both cohorts for a detected class species, red arrows indicate an overall increase and blue arrows indicate an overall decrease. Double-headed arrows indicate a mix of increased and decreased species. Black boxes indicate the species that were undetected. CDP-DG, cytidine diphosphate-diacylglycerol; Cer, ceramide; CL, cardiolipin; DG, diacylglycerol; FFA, free fatty acid; GM3, GM3 ganglioside; GlcCer, glucosylceramide; LacCer, lactosylceramide; LPA, lysophosphatidic acid; LPC, lysophosphatidylcholine; LPE, lysophosphatidylethanolamine; LPG, lysophosphatidylglycerol; LPI, lysophosphatidylinositol; LPS, lysophosphatidylserine; MG, monoacylglycerol; PA, phosphatidic acid; PC, phosphatidylcholine; PE, phosphatidylethanolamine; PG, phosphatidylglycerol; PGP, phosphatidylglycerophosphate; PI, phosphatidylinositol; PS, phosphatidylserine; SM, sphingomyelin; TG, triacylglycerol. [Created in BioRender. Culp-Hill, R. (2025) https://BioRender.com/fyq73z7.]

Lipid metabolism is a highly dynamic and deeply interconnected network of species. Each subclass comprises many individual species, in some cases over 750. We identified several lipid classes of interest (phospholipids, glycerides, ceramides, fatty acids, and gangliosides) across two independent cohorts designed to represent the population experiencing signs and symptoms of ovarian cancer. These classes have strong potential for detection of ovarian cancer in this complex population, with further implications in other diseases. Figure 4 presents a metabolic map of lipid interconversion in ovarian cancer, with arrows depicting the direction in which the sum of all species within a class was significantly altered. In some cases, although the sum of all species remained unchanged, further analysis revealed significant shifts in individual species, with some increasing and others decreasing. Lipid reprogramming has been well documented in the context of cancer, influencing signaling, membrane remodeling, and cellular metabolism, and these alterations are reflected in serum (17). This figure provides a framework to understand lipidomic alterations in the context of ovarian cancer, comparing serum across all ovarian cancer stages and a range of histologic classifications with a clinically complex range of controls representing the symptomatic population.

Our work shows the potential of lipids as diagnostic biomarkers in a clinical diagnostic assay through independent univariate analysis. Through ML-based multiomic modeling in a proof-of-concept study, we determined that a novel combination of lipids and a few proteins—fewer than 20 biomarkers in total—achieves AUCs of 92% in controls versus ovarian cancer and 88% in controls versus early-stage ovarian cancer in the holdout cohort, of which 75% of the samples were from a unique prospectively collected study of women with symptoms of ovarian cancer. The cohorts were designed with deliberate enrichment for biologically challenging cases, such as benign adnexal masses and clinically advanced GI patients. To our knowledge, they are the first of their kind to enable molecular characterization of specimens representing the VAS population and incorporate serum from early and late-stage ovarian cancer, benign adnexal masses, GI disorders, and otherwise healthy individuals as baseline. This is also the first report of global lipid profiling on this highly complex symptomatic population: previous research has focused on ovarian cancer versus healthy individuals, patients with ovarian cancer undergoing treatment for analyses of prognostic measures, and ovarian cancer versus adnexal masses in individuals scheduled for surgery (18). Our findings indicate that lipids could be leveraged for detection of early-stage disease, enhancing clinical performance in distinguishing all ovarian cancer stages and subtypes from a wide range of clinically relevant controls when combined with other biomarker classes.

The combination of lipid and protein biomarkers enables robust detection across the disease spectrum, making this method efficient, non-invasive, and compatible with existing clinical workflows. In contrast to DNA-based tests, which are unproven in early-stage ovarian cancer (42, 49), our lipid-based method is streamlined, requiring a single extraction reagent and providing rapid sample processing (minutes per sample). Our approach also requires less than 500 μL serum compared with milliliters of plasma needed for ctDNA-based approaches.

Although the compelling data and model presented here showcase the groundbreaking potential of multiomics for early-stage ovarian cancer detection and transformation of the ovarian cancer landscape, this approach is not without limitations, which we aim to address in future work. First, for proteins, when applying the commonly used clinical cutoff of 35 U/mL for CA125 alone, the observed sensitivity was 76.2% for overall ovarian cancer and 65.2% for early-stage ovarian cancer, a performance consistent with clinical reports. However, specificity among controls was higher than expected at 92.3%. This is likely due to the use of a Research Use Only (RUO) assay for determining CA125 values. Indeed, when comparing results from the RUO assay used here with those available in the specimen clinical history, values were well correlated, but RUO results showed a negative bias.

Second, obtaining samples from patients diagnosed with ovarian cancer across stages and subtypes is challenging because of low prevalence; we were unable to obtain details on pre-analytic factors, including blood collection and processing conditions. Additionally, we did not have control over fasting status, body mass index, and detailed information on comorbidities, including inflammatory diseases and various medications (statins, β-blockers, and hormonal medications), which can influence lipid profiles. Therefore, it is not surprising that a large number (>2,800) of lipid features did not overlap between the cohorts. However, we were able to profile similarities in lipid classes and identify potential biomarkers that will need to be validated for identification and quantitation with internal standards using a targeted approach. Another caveat is that specimens from our cohorts are relatively homogeneous in ethnicity, and in the United States, ovarian cancer is most prevalent in non-Hispanic White women (50). Interestingly, CA125 levels are known to differ by ethnicity (51), so further testing will aim to include a greater ethnic representation to determine whether the lipid signatures remain consistent.

A third caveat to our study is that untargeted and targeted MS each has distinct advantages for lipidomics analysis. Targeted analysis focuses on quantification of a limited number of predefined lipid species using internal standards, whereas untargeted methods putatively identify a broad spectrum of lipid species, some of which could be novel biomarkers. The untargeted strategy allowed us to cast as wide a net as possible to identify novel lipid biomarkers rather than being limited to a smaller number of species analyzed within a targeted analysis (26). However, untargeted MS standardization is fundamentally challenging (52) as normalizing across cohorts or batches is inherently difficult without targeting specific compounds and/or obtaining internal standards for each target. Here, 50 samples were processed with both cohorts to enable the batch correction portion of the normalization process that was critical for cross-cohort model performance. Although a shared panel of reference samples was an effective normalization tool for these studies, it is not a long-term, scalable solution. By using industry-standard targeted platforms such as triple quadrupole-based MS, along with appropriate reference standards, we can report the identity of lipid species with high confidence, including structural class, acyl chain composition, and, where applicable, positional or isomeric variants. Given the absence of this analytic framework, the data presented here should be viewed as proof of concept, with full acknowledgment of the associated limitations. Our future work is focused on migration of key features to a targeted assay. Subsequent analytic and clinical validation is required for confirmation of their identities and to accurately quantify them for diagnostic application.

In closing, we demonstrate that our novel ML-based proof-of-concept multiomic model achieves high AUCs, even in early-stage cases, and remains robust despite differences in cohort demographics, sources, and clinical characteristics. The integration of lipid and protein biomarkers is adaptable across independent patient populations, highlighting its potential for broad application in ovarian cancer detection. ML and multiomic analysis is a novel approach with potential for improved performance over current methods that can guide future researchers in the study of various diseases and fill the gap of unmet clinical need, shortening time to diagnosis and improving patient outcomes. These findings provide proof of concept that lipids may serve as diagnostic biomarkers for early ovarian cancer within the clinically complex symptomatic population, particularly when applied in a multiomic approach.

## Supplementary Material

Supplemental Figure 1 Supplemental Figure 1. Discovery-based lipidomics feature filtering strategy

Supplemental Figure 2 Supplemental Figure 2. Cohort 1 PLSDA and heatmaps of top 50 gangliosides by ANOVA comparing controls to OC and early-stage OC

Supplemental Figure 3 Supplemental Figure 3. Cohort 2 PLSDA and heatmaps of top 50 gangliosides by ANOVA comparing controls to OC and early-stage OC

Supplemental Figure 4 Supplemental Figure 4. Global Serum Lipid Profile in Cohort 1 and Cohort 2

Supplemental Figure 5 Supplemental Figure 5. Heatmaps of Top 100 Lipid Features by ANOVA across Cohorts and Comparisons

Supplemental Figure 6 Supplemental Figure 6. PLSDA Variable Importance Scores across Cohorts and Comparisons

Supplemental Figure 7 Supplemental Figure 7. PLSDA and heatmaps of top 100 lipid features by ANOVA across cohorts comparing controls to early-stage OC and late-stage OC

Supplemental Table 1 Supplemental Table 1. Cohort Disease States

Supplemental Table 2 Supplemental Table 2. Cohort Demographic Details

Supplemental Table 3 Supplemental Table 3. Normalization Subset of Cohort 1
