# Supplementary material for: Utilizing Serum-Derived Lipidomics with Protein Biomarkers and Machine Learning for Early Detection of Ovarian Cancer in the Symptomatic Population
Source: Cancer Res Commun. 2025 Sep 4;5(9):1516–29. doi: 10.1158/2767-9764.CRC-25-0140 (PMC12409608; doi:10.1158/2767-9764.CRC-25-0140)
Supplement: Supplemental Figure 1 — Discovery-based lipidomics feature filtering strategy [file crc-25-0140_supplemental_figure_1_suppsf1.pdf]

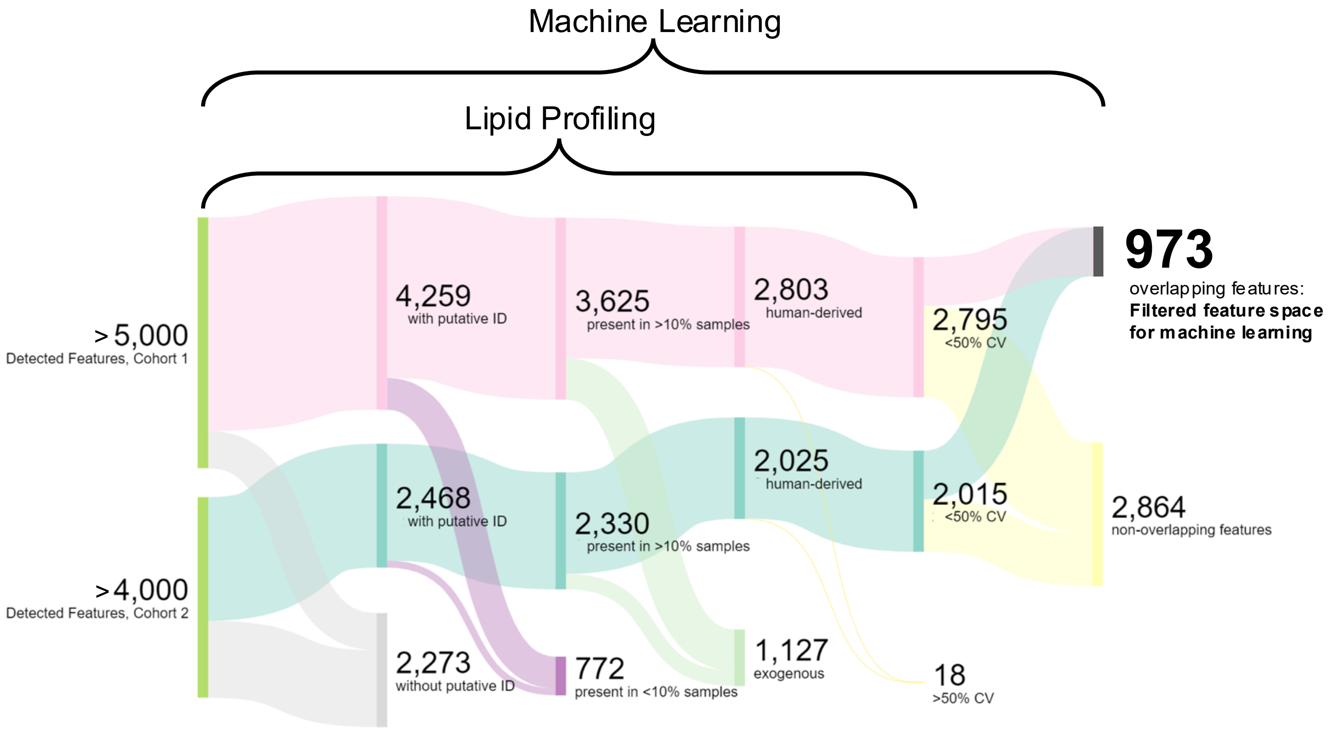

Supplemental Figure 1. Discovery-based lipidomics feature filtering strategy. Schematic illustrating the systematic feature selection process applied to two independent cohorts. The feature spaces underwent the following filtering steps: (1) exclusion of features without a putative ID through library matching, (2) exclusion of features present in less than 10% of samples within a cohort, (3) exclusion of features of exogenous origin, including drugs, synthetic compounds, dietary metabolites, and non-microbiome bacterial products, and (4) exclusion of features exhibiting high technical variability (CV > 50%) across the technical QC sample. Only these steps were used to filter data for lipid profiling analysis. The feature space was then (5) filtered to include only those features detected across both cohorts prior to inclusion in the feature space for machine learning.
