# Supplementary material for: Utilizing Serum-Derived Lipidomics with Protein Biomarkers and Machine Learning for Early Detection of Ovarian Cancer in the Symptomatic Population
Source: Cancer Res Commun. 2025 Sep 4;5(9):1516–29. doi: 10.1158/2767-9764.CRC-25-0140 (PMC12409608; doi:10.1158/2767-9764.CRC-25-0140)
Supplement: Supplemental Figure 2 — Cohort 1 PLSDA and heatmaps of top 50 gangliosides by ANOVA comparing controls to OC and early-stage OC [file crc-25-0140_supplemental_figure_2_suppsf2.pdf]

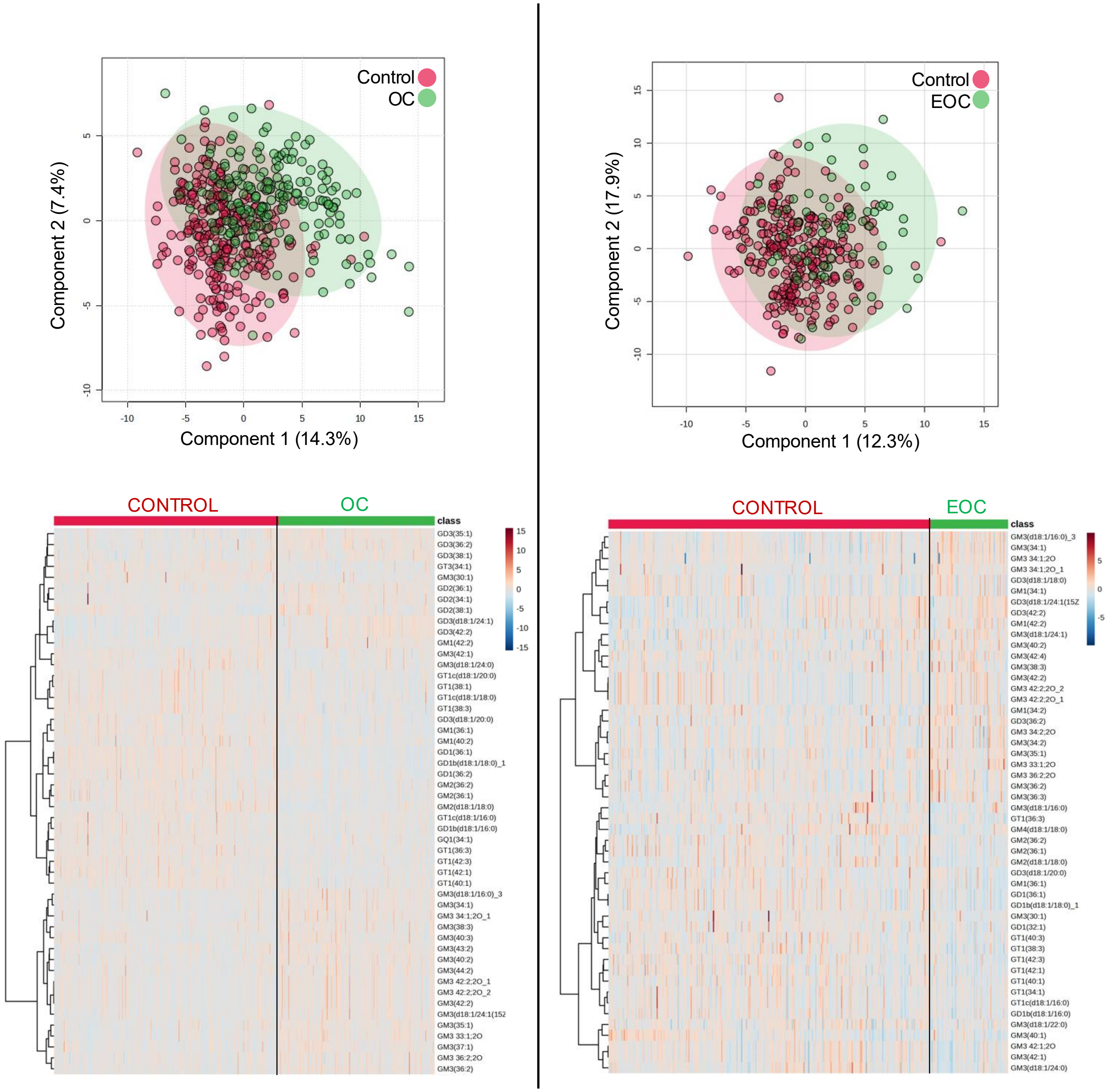

<u>Supplemental Figure 2. Cohort 1 PLSDA and heatmaps of top 50 gangliosides by ANOVA comparing controls to OC and early-stage OC.</u> Heatmaps depicting the top 50 gangliosides by ANOVA for Cohort 1. Features are grouped by hierarchical clustering. Color scale reflects relative changes, with red indicating higher abundance and blue indicating lower abundance. EOC = early-stage ovarian cancer (stages I/II), OC = all stages of ovarian cancer (stages I/II/III/IV).
