# Supplementary material for: Utilizing Serum-Derived Lipidomics with Protein Biomarkers and Machine Learning for Early Detection of Ovarian Cancer in the Symptomatic Population
Source: Cancer Res Commun. 2025 Sep 4;5(9):1516–29. doi: 10.1158/2767-9764.CRC-25-0140 (PMC12409608; doi:10.1158/2767-9764.CRC-25-0140)
Supplement: Supplemental Figure 4 — Global Serum Lipid Profile in Cohort 1 and Cohort 2 [file crc-25-0140_supplemental_figure_4_suppsf4.pdf]

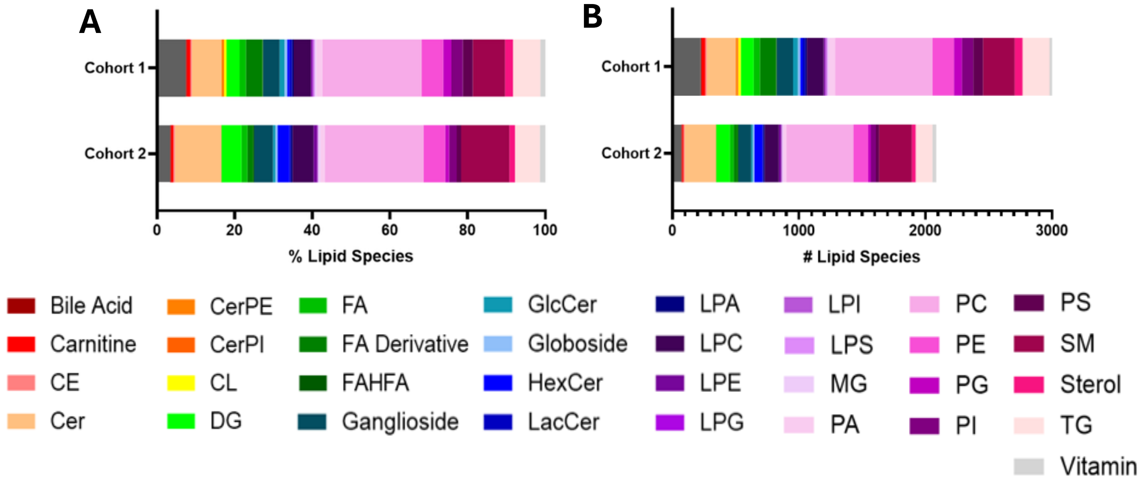

Supplemental Figure 4. Global Serum Lipid Profile in Cohort 1 and Cohort 2.
A. Lipid class assignments as a percentage of the total detected lipids after QC filtering. B. Total number of lipid class assignments after QC filtering.

CE: Cholesteryl Ester; **CerPE**: Ceramide Phosphoethanolamine;
CerPI: Ceramide Phosphoinositol; **CL**: Cardiolipin; **DG**: Diacylglycerol;
FA: Fatty Acid; **FA Derivative**: Fatty Acid Derivative;
FAHFA: Fatty Acid Esters of Hydroxy Fatty Acids; **GlcCer**: Glucosylceramide;
HexCer: Hexosylceramide; **LacCer**: Lactosylceramide;
LPA: Lysophosphatidic Acid; **LPC**: Lysophosphatidylcholine;
LPE: Lysophosphatidylethanolamine; **LPG**: Lysophosphatidylglycerol;
LPI: Lysophosphatidylinositol; **LPS**: Lysophosphatidylserine;
MG: Monoacylglycerol; **PA**: Phosphatidic Acid; **PC**: Phosphatidylcholine;
PE: Phosphatidylethanolamine; **PG**: Phosphatidylglycerol; **PI**: Phosphatidylinositol;
PS: Phosphatidylserine; **SM**: Sphingomyelin; **TG**: Triacylglycerol
