# Supplementary material for: Utilizing Serum-Derived Lipidomics with Protein Biomarkers and Machine Learning for Early Detection of Ovarian Cancer in the Symptomatic Population
Source: Cancer Res Commun. 2025 Sep 4;5(9):1516–29. doi: 10.1158/2767-9764.CRC-25-0140 (PMC12409608; doi:10.1158/2767-9764.CRC-25-0140)
Supplement: Supplemental Figure 5 — Heatmaps of Top 100 Lipid Features by ANOVA across Cohorts and Comparisons [file crc-25-0140_supplemental_figure_5_suppsf5.pdf]

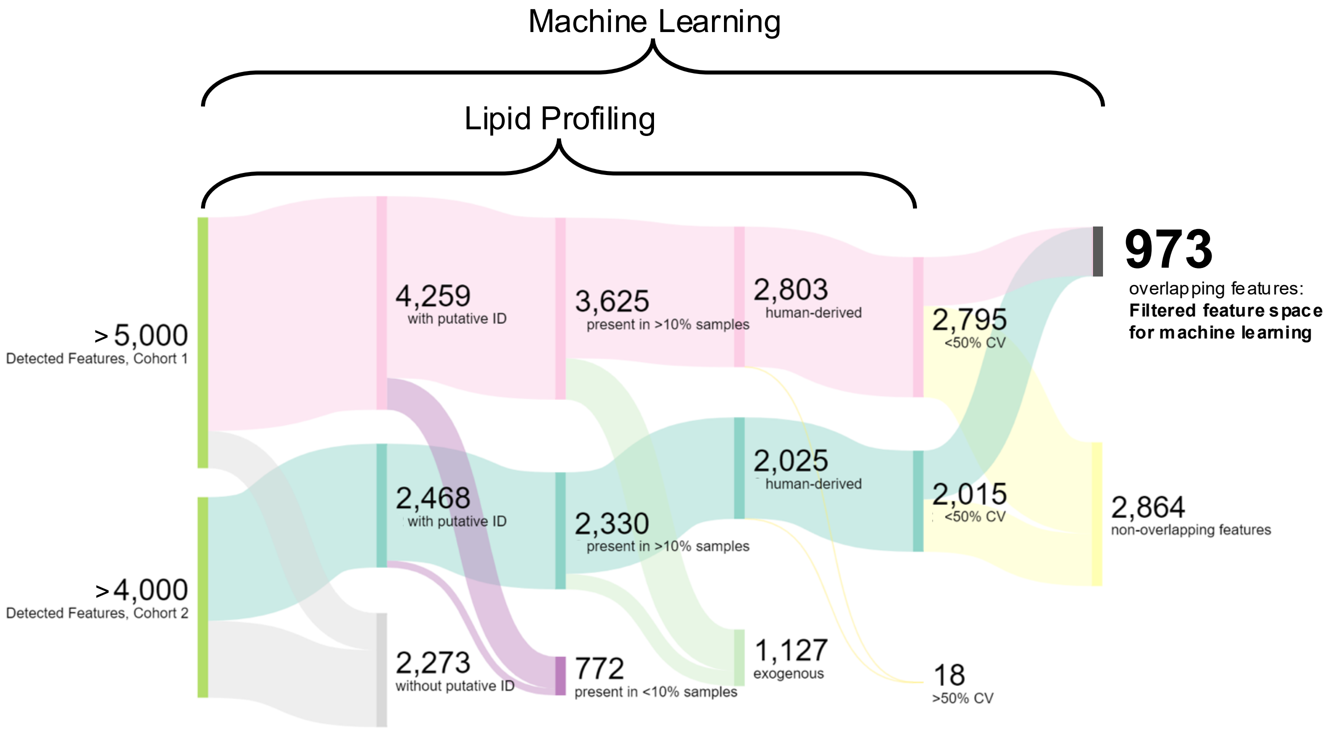

<u>Supplemental Figure 1. Discovery-based lipidomics feature filtering strategy.</u> Schematic illustrating the systematic feature selection process applied to two independent cohorts. The feature spaces underwent the following filtering steps: (1) exclusion of features without a putative ID through library matching, (2) exclusion of features present in less than 10% of samples within a cohort, (3) exclusion of features of exogenous origin, including drugs, synthetic compounds, dietary metabolites, and non-microbiome bacterial products, and (4) exclusion of features exhibiting high technical variability (CV > 50%) across the technical QC sample. Only these steps were used to filter data for lipid profiling analysis. The feature space was then (5) filtered to include only those features detected across both cohorts prior to inclusion in the feature space for machine learning.

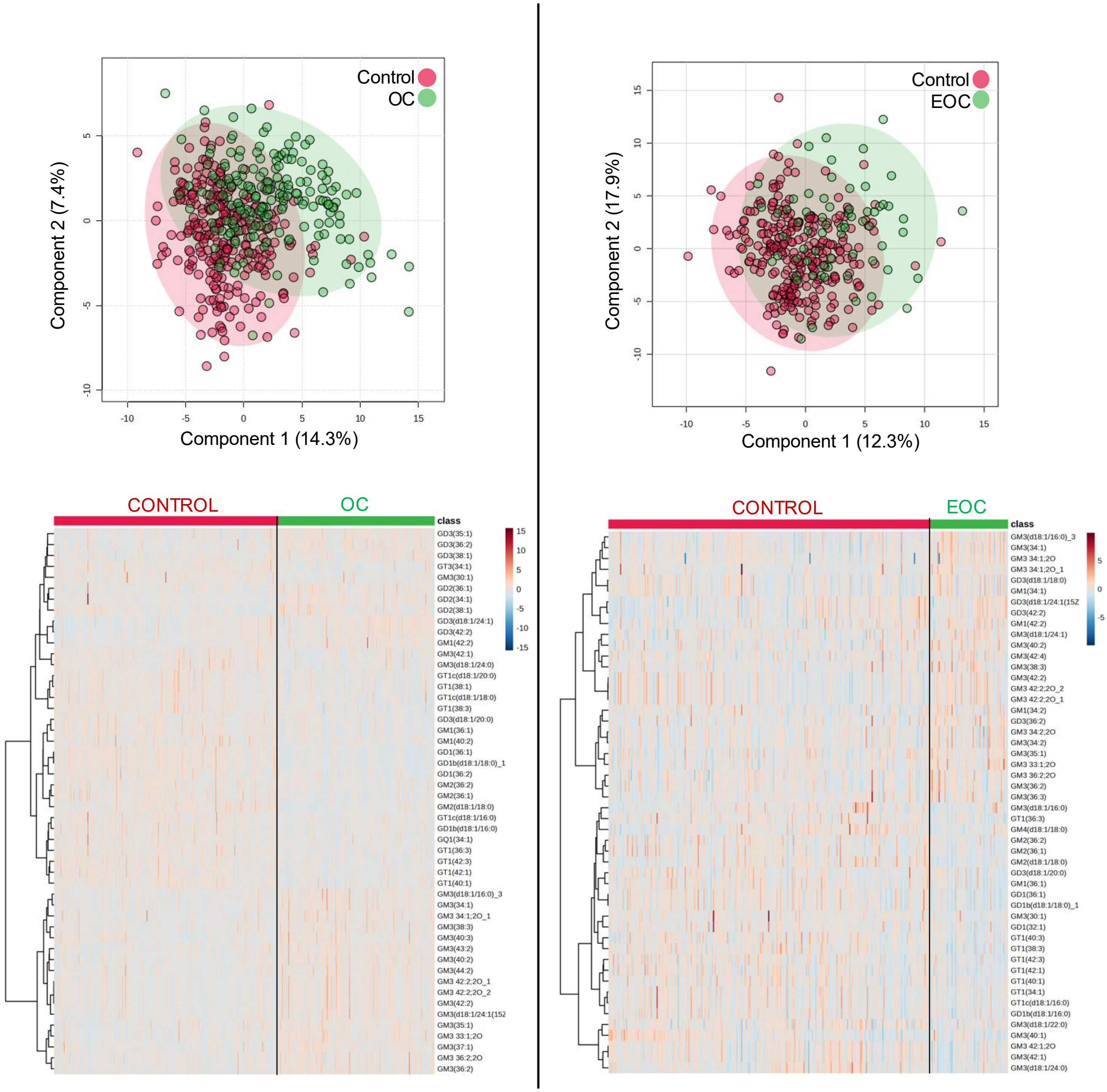

Supplemental Figure 2. Cohort 1 PLSDA and heatmaps of top 50 gangliosides by ANOVA comparing controls to OC and early-stage OC. Heatmaps depicting the top 50 gangliosides by ANOVA for Cohort 1. Features are grouped by hierarchical clustering. Color scale reflects relative changes, with red indicating higher abundance and blue indicating lower abundance. EOC = early-stage ovarian cancer (stages I/II), OC = all stages of ovarian cancer (stages I/II/III/IV).

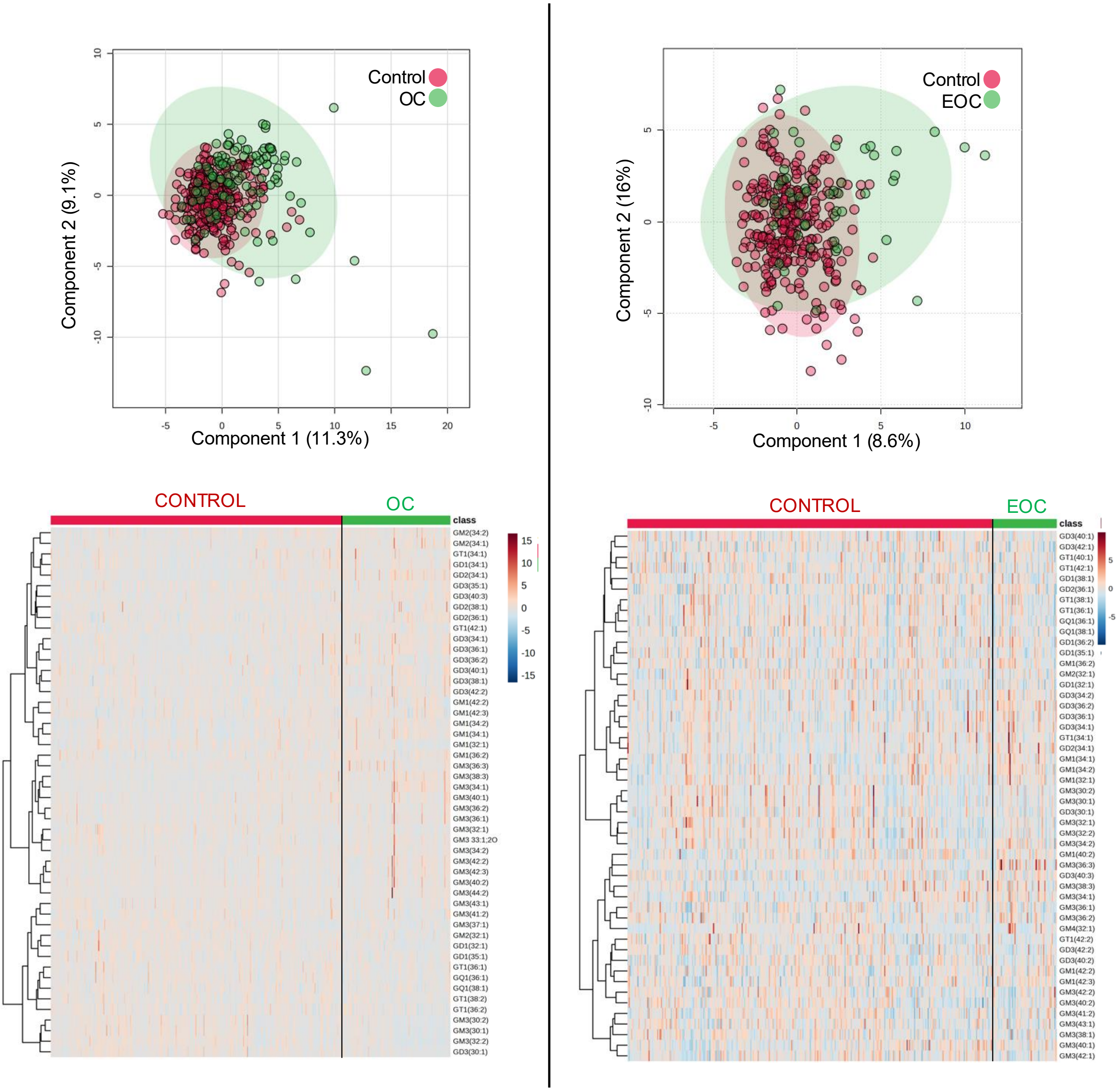

Supplemental Figure 3. Cohort 2 PLSDA and heatmaps of top 50 gangliosides by ANOVA comparing controls to OC and early-stage OC. Heatmaps depicting the top 50 gangliosides by ANOVA for Cohort 2. Features are grouped by hierarchical clustering. Color scale reflects relative changes, with red indicating higher abundance and blue indicating lower abundance. EOC = early-stage ovarian cancer (stages I/II), OC = all stages of ovarian cancer (stages I/II/III/IV).

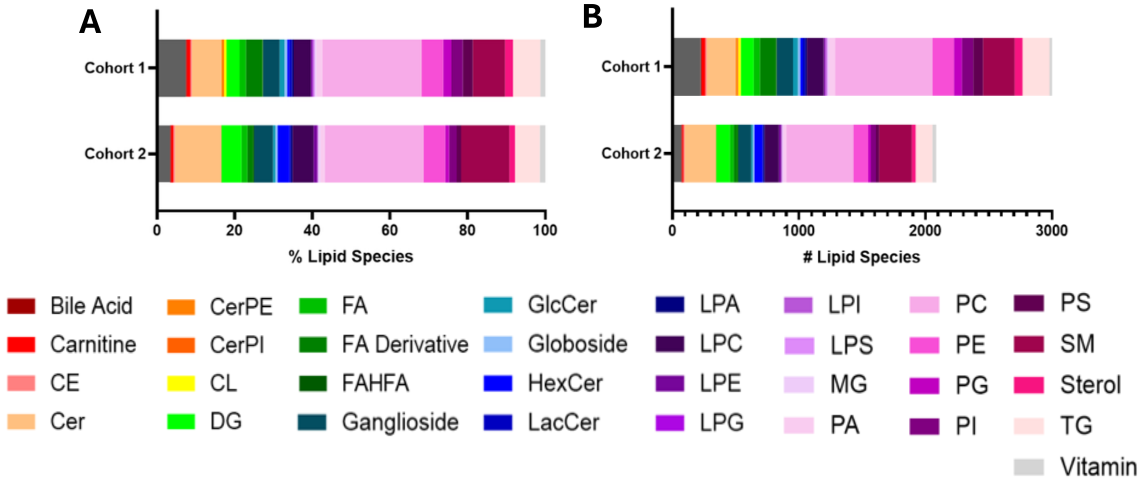

Supplemental Figure 4. Global Serum Lipid Profile in Cohort 1 and Cohort 2.
A. Lipid class assignments as a percentage of the total detected lipids after QC filtering. B. Total number of lipid class assignments after QC filtering.

CE: Cholesteryl Ester; **CerPE**: Ceramide Phosphoethanolamine;
CerPI: Ceramide Phosphoinositol; **CL**: Cardiolipin; **DG**: Diacylglycerol;
FA: Fatty Acid; **FA Derivative**: Fatty Acid Derivative;
FAHFA: Fatty Acid Esters of Hydroxy Fatty Acids; **GlcCer**: Glucosylceramide;
HexCer: Hexosylceramide; **LacCer**: Lactosylceramide;
LPA: Lysophosphatidic Acid; **LPC**: Lysophosphatidylcholine;
LPE: Lysophosphatidylethanolamine; **LPG**: Lysophosphatidylglycerol;
LPI: Lysophosphatidylinositol; **LPS**: Lysophosphatidylserine;
MG: Monoacylglycerol; **PA**: Phosphatidic Acid; **PC**: Phosphatidylcholine;
PE: Phosphatidylethanolamine; **PG**: Phosphatidylglycerol; **PI**: Phosphatidylinositol;
PS: Phosphatidylserine; **SM**: Sphingomyelin; **TG**: Triacylglycerol

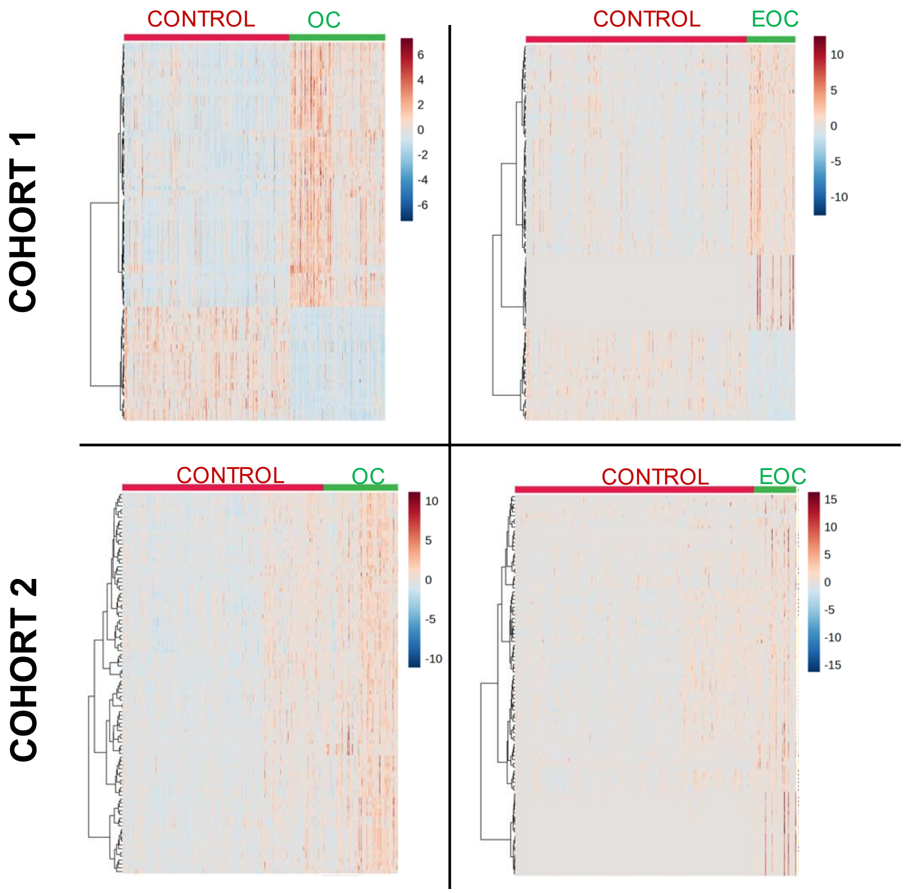

<u>Supplemental Figure 5. Heatmaps of Top 100 Lipid Features by ANOVA across Cohorts and Comparisons.</u> Heatmaps depicting the top 100 features by ANOVA for Cohort 1 (top) and Cohort 2 (bottom). Comparisons include control vs. OC cases (left) and control vs. early-stage OC cases (right). Features are grouped by hierarchical clustering. Color scale reflects relative changes, with red indicating higher abundance and blue indicating lower abundance.

CONTROL V. OC

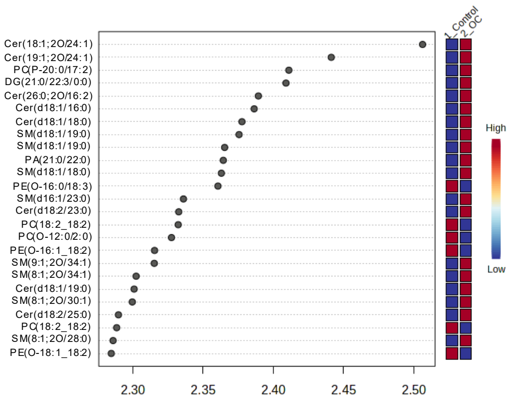

COHORT 1

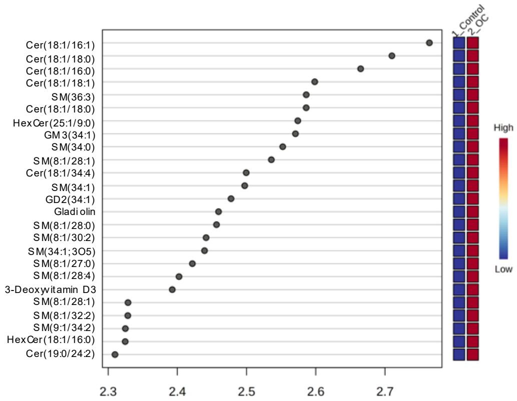

COHORT 2

CONTROL V. EARLY-STAGE OC

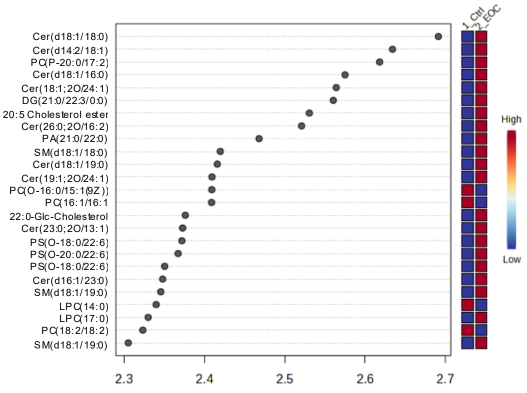

COHORT 1

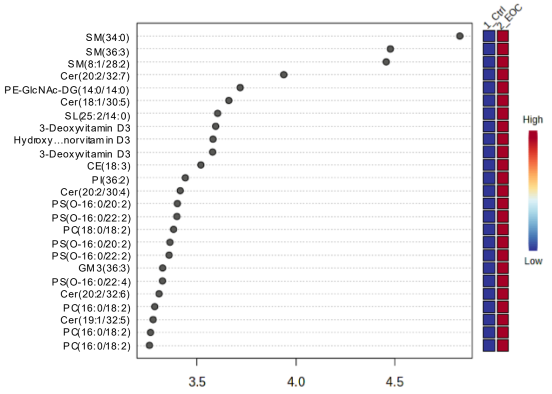

COHORT 2

<u>Supplemental Figure 6. PLSDA variable importance scores across cohorts and comparisons.</u> Variable Importance in Projection (VIP) scores from PLSDA highlighting the top 25 most discriminative features for each cohort, comparing controls vs. OC and early-stage OC. The VIP plot ranks features based on contribution to the PLSDA model, with higher scores indicating greater importance in distinguishing sample groups. Features exceeding the common VIP threshold of 2.0 are considered highly influential in driving group separation.

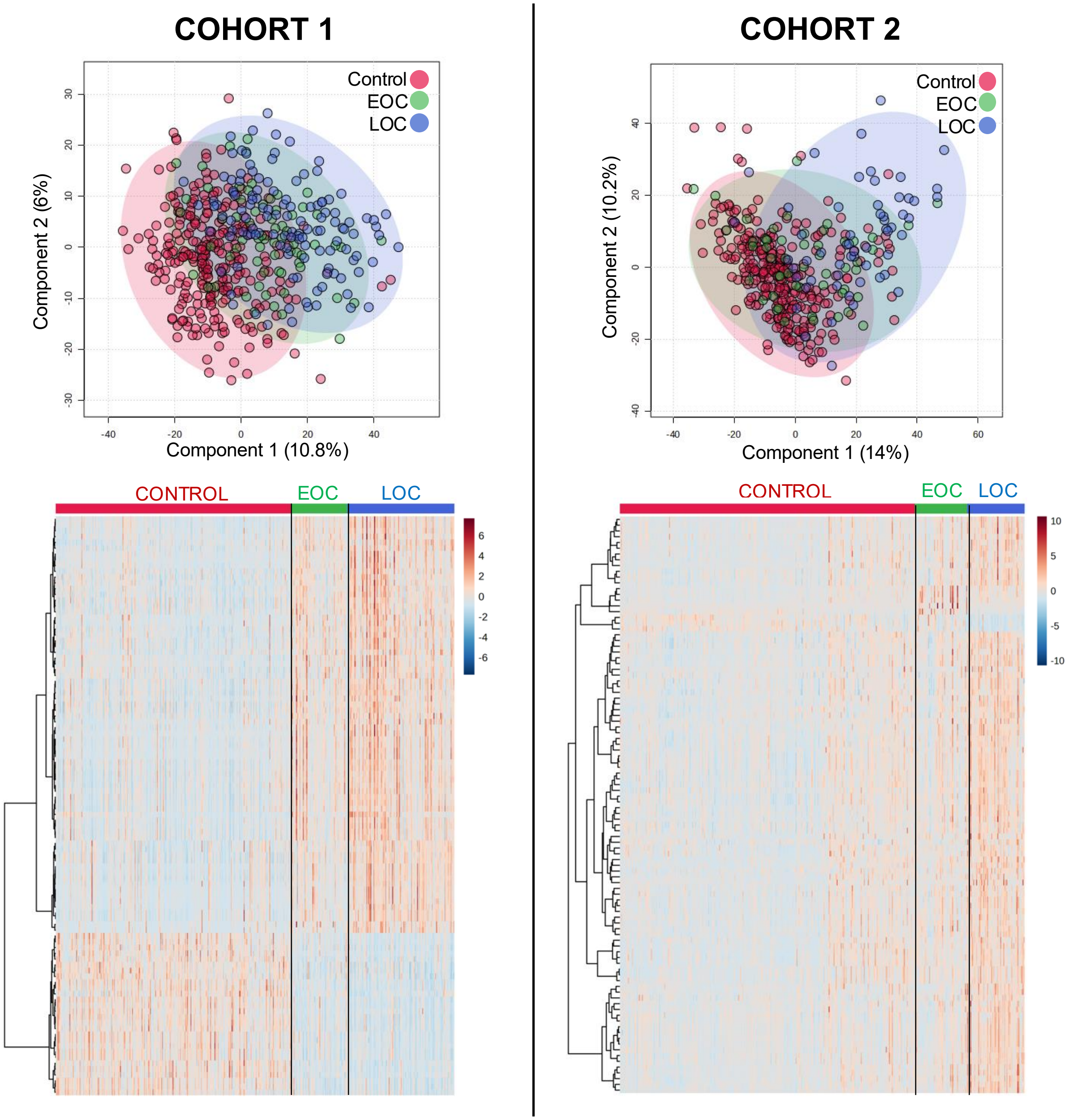

Supplemental Figure 7. PLSDA and heatmaps of top 100 lipid features by ANOVA across cohorts comparing controls to early-stage OC and late-stage OC. Heatmaps depicting the top 100 features by ANOVA for Cohort 1 (left) and Cohort 2 (right). Features are grouped by hierarchical clustering. Color scale reflects relative changes, with red indicating higher abundance and blue indicating lower abundance. EOC = early-stage ovarian cancer (stages I/II), LOC = late-stage ovarian cancer (stages III/IV).
