# Supplementary material for: Utilizing Serum-Derived Lipidomics with Protein Biomarkers and Machine Learning for Early Detection of Ovarian Cancer in the Symptomatic Population
Source: Cancer Res Commun. 2025 Sep 4;5(9):1516–29. doi: 10.1158/2767-9764.CRC-25-0140 (PMC12409608; doi:10.1158/2767-9764.CRC-25-0140)
Supplement: Supplemental Figure 6 — PLSDA Variable Importance Scores across Cohorts and Comparisons [file crc-25-0140_supplemental_figure_6_suppsf6.pdf]

CONTROL V. OC

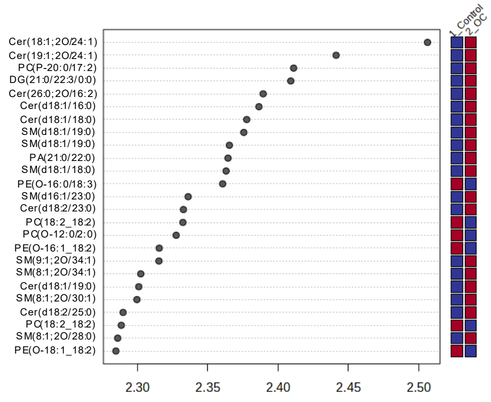

COHORT 1

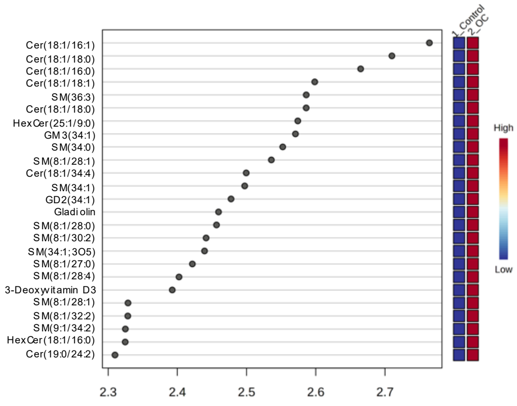

COHORT 2

CONTROL V. EARLY-STAGE OC

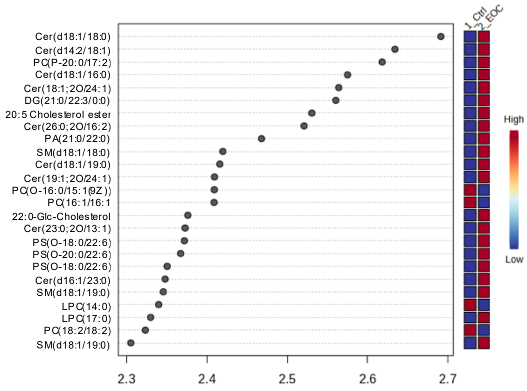

COHORT 1

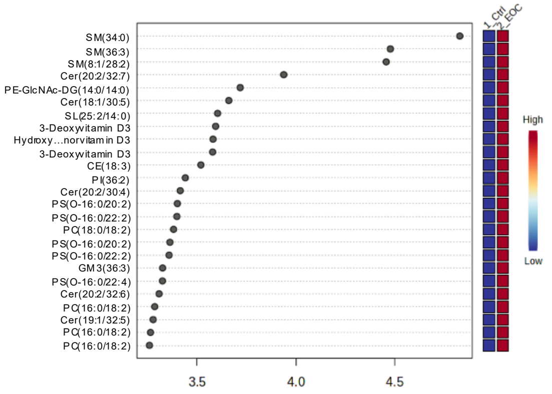

COHORT 2

<u>Supplemental Figure 6. PLSDA Variable Importance Scores across Cohorts and Comparisons.</u> Variable Importance in Projection (VIP) scores from PLSDA highlighting the top 25 most discriminative features for each cohort, comparing controls vs. OC and early-stage OC. The VIP plot ranks features based on contribution to the PLSDA model, with higher scores indicating greater importance in distinguishing sample groups. Features exceeding the common VIP threshold of 2.0 are considered highly influential in driving group separation.
