# Supplementary material for: Utilizing Serum-Derived Lipidomics with Protein Biomarkers and Machine Learning for Early Detection of Ovarian Cancer in the Symptomatic Population
Source: Cancer Res Commun. 2025 Sep 4;5(9):1516–29. doi: 10.1158/2767-9764.CRC-25-0140 (PMC12409608; doi:10.1158/2767-9764.CRC-25-0140)
Supplement: Supplemental Figure 7 — PLSDA and heatmaps of top 100 lipid features by ANOVA across cohorts comparing controls to early-stage OC and late-stage OC [file crc-25-0140_supplemental_figure_7_suppsf7.pdf]

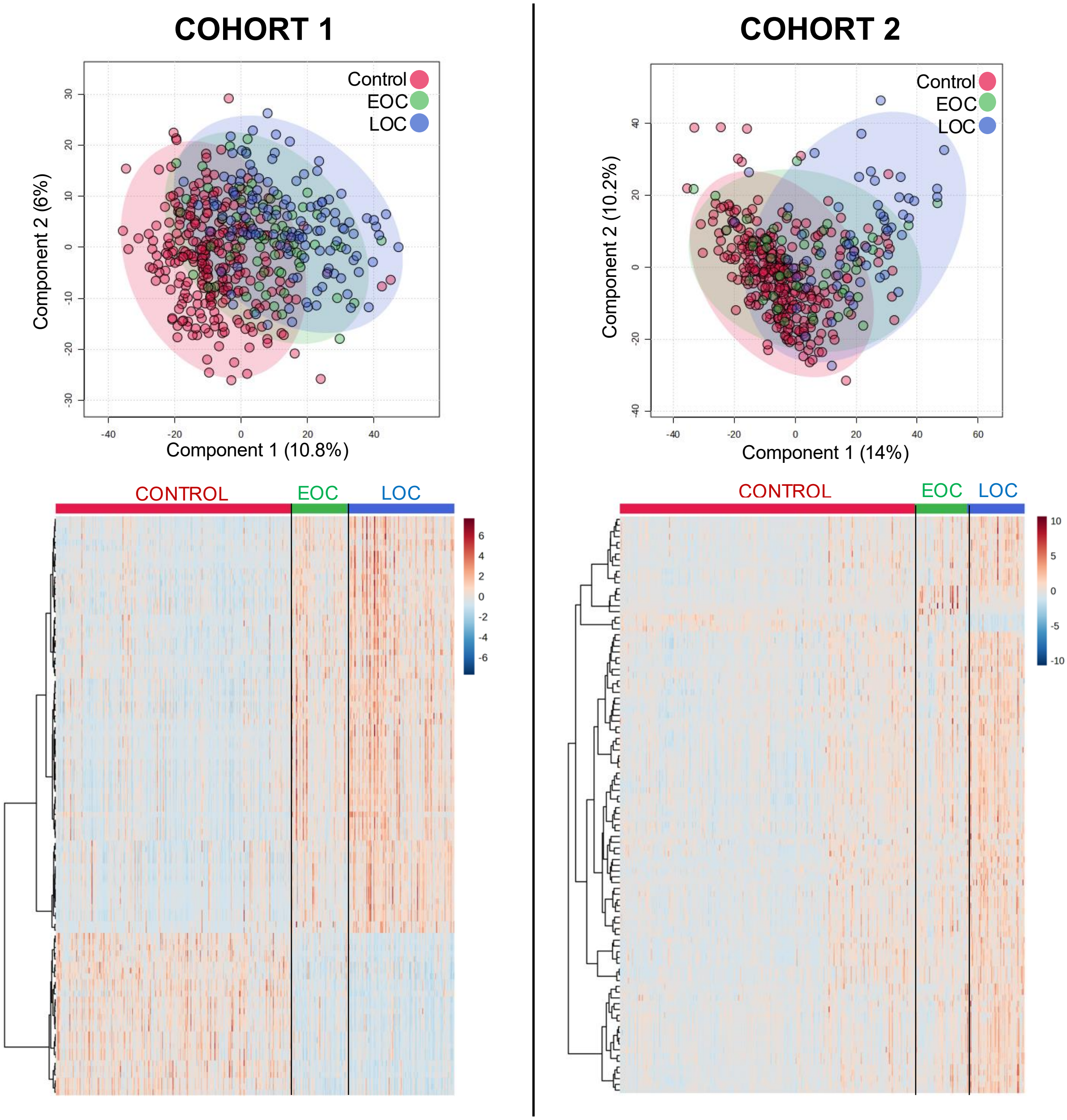


**<u>Supplemental Figure 7. PLSDA and heatmaps of top 100 lipid features by ANOVA across cohorts comparing controls to early-stage OC and late-stage OC</u>**. Heatmaps depicting the top 100 features by ANOVA for Cohort 1 (left) and Cohort 2 (right). Features are grouped by hierarchical clustering. Color scale reflects relative changes, with red indicating higher abundance and blue indicating lower abundance. EOC = early-stage ovarian cancer (stages I/II), LOC = late-stage ovarian cancer (stages III/IV).
