# Supplementary material for: Utilizing Serum-Derived Lipidomics with Protein Biomarkers and Machine Learning for Early Detection of Ovarian Cancer in the Symptomatic Population
Source: Cancer Res Commun. 2025 Sep 4;5(9):1516–29. doi: 10.1158/2767-9764.CRC-25-0140 (PMC12409608; doi:10.1158/2767-9764.CRC-25-0140)
Supplement: Supplemental Table 1 — Cohort Disease States [file crc-25-0140_supplemental_table_1_suppst1.pdf]

Supplemental Table 1. Cohort Disease States. Descriptions and examples of diagnoses included in the study design.

Term	Description	Example
Serous	The most common subtype of epithelial ovarian cancer that originates in the serous membrane covering fallopian tubes	serous adenocarcinoma
Mucinous	A subtype of epithelial ovarian cancer characterized by tumors containing mucus-producing cells	mucinous ovarian carcinoma
Endometrioid	A subtype of epithelial ovarian cancer that histologically resembles endometrial tissue with glands and other features similar to endometrial cancer	endometrioid adenocarcinoma
Clear Cell	A subtype of epithelial ovarian cancer named for its cells' uniquely transparent appearance under a microscope	high grade clear cell ovarian carcinoma
Mixed	Mixed epithelial ovarian cancer arises when two or more distinct cell types from different histological subtypes of ovarian cancer are present	endometrioid ovarian carcinoma with mucinous differentiation
Non-Epithelial	Rare, diverse subtypes of ovarian cancer that do not originate from epithelial cells including germ cell tumors and sex-cord stromal tumors	granulosa cell tumor
Not Reported	In some cases, ovarian cancer pathology reports describing histological findings were not available	unknown histology
Benign: mass	A non-cancerous growth of tissue. These masses are generally slow growing, do not invade nearby tissues, and do not spread to other parts of the body	leiomyoma, fibroma, or cystadenofibroma
Benign: cyst	A sac-like pocket of membranous tissue that contains fluid, air, or other substances	dermoid cyst, serous cystadenoma, or endometrioma
Benign: growth	Refers to an abnormal increase in tissue size, which does not invade nearby tissue or metastasize, but it may require monitoring or removal	typical hyperplasia or thecofibroma
Benign: other	Symptomatic but non-complex benign diagnoses with unclear or unavailable pathology	unknown histology
GI Disorder	Gastrointestinal (GI) disorders affect the digestive system which present a variety of symptoms including abdominal discomfort	diverticulitis or active chronic gastritis
Normal	A normal disease state in the context of this study refers to patients with no known diagnosed disease	no active clinical diagnosis
