# Supplementary material for: Utilizing Serum-Derived Lipidomics with Protein Biomarkers and Machine Learning for Early Detection of Ovarian Cancer in the Symptomatic Population
Source: Cancer Res Commun. 2025 Sep 4;5(9):1516–29. doi: 10.1158/2767-9764.CRC-25-0140 (PMC12409608; doi:10.1158/2767-9764.CRC-25-0140)
Supplement: Supplemental Table 2 — Cohort Demographic Details [file crc-25-0140_supplemental_table_2_suppst2.pdf]

Supplemental Table 2. Cohort Demographic Details. Detailed breakout of relevant clinical details for both cohorts: age, race/ethnicity, histology, and stage.

	Cohort #1 (PT): N = 487				Cohort #2 (JT): N = 402				Total: N = 889			
	OC Cases		Controls		OC Cases		Controls		OC Cases		Controls	
	n = 187		n = 300		n = 109		n = 293		n = 296		n = 593	
	n	%	n	%	n	%	n	%	n	%	n	%
**Age**												
<50	34	18.2	122	40.7	28	25.7	35	11.9	62	20.9	157	24.6
50-59	64	34.2	97	32.3	39	35.8	48	16.4	103	34.8	145	27.9
60-69	58	31.0	52	17.3	24	22.0	68	23.2	82	27.7	120	22.7
70+	30	16.0	22	7.3	18	16.5	142	48.5	48	16.2	164	23.8
Unknown	1	0.5	7	2.3	0	0.0	0	0.0	1	0.3	7	0.9
Mean (SD)	58.8 (11.4)		50.9 (13.2)		57.6 (12.1)		66.5 (13.4)		58.3 (11.7)		58.7 (15.4)	
**Race/Ethnicity**												
African American	0	0.0	2	0.7	1	0.9	12	4.1	1	0.3	14	2.4
Arab	0	0.0	0	0.0	0	0.0	1	0.3	0	0.0	1	0.2
Asian	0	0.0	0	0.0	6	5.5	19	6.5	6	2.0	19	3.2
Caucasian	71	38.0	233	77.7	101	92.7	243	82.9	172	58.1	476	80.3
Hispanic	24	12.8	2	0.7	1	0.9	0	0.0	25	8.4	2	0.3
Unreported	92	49.2	63	21.0	0	0.0	18	6.1	92	31.1	81	13.7
**Histology**												
Serous	121	64.7			61	56.0			182	61.5		
Mucinous	16	8.6			11	10.1			27	9.1		
Endometrioid	15	8.0			18	16.5			33	11.1		
Clear Cell	12	6.4			11	10.1			23	7.8		
Mixed	4	2.1			1	0.9			5	1.7		
Non-Epithelial	1	0.5			1	0.9			2	0.7		
Not Reported	18	9.6			6	5.5			24	8.1		
Benign: mass			51	17.0			28	9.6			79	13.3
Benign: growth			13	4.3			16	5.5			29	4.9
Benign: cyst			93	31.0			39	13.3			132	22.3
Benign: other			11	3.7			2	0.7			13	2.2
GI Disorder			50	16.7			0	0.0			50	8.4
Normal			82	27.3			208	71.0			290	48.9
**Stage**												
I	48	25.7			40	36.7			88	29.7		
II	24	12.8			12	11.0			36	12.2		
III	97	51.9			41	37.6			138	46.6		
IV	16	8.6			16	14.7			32	10.8		
Unstaged	2	1.1			0	0.0			2	0.7		
