# Supplementary material for: Utilizing Serum-Derived Lipidomics with Protein Biomarkers and Machine Learning for Early Detection of Ovarian Cancer in the Symptomatic Population
Source: Cancer Res Commun. 2025 Sep 4;5(9):1516–29. doi: 10.1158/2767-9764.CRC-25-0140 (PMC12409608; doi:10.1158/2767-9764.CRC-25-0140)
Supplement: Supplemental Table 3 — Normalization Subset of Cohort 1 [file crc-25-0140_supplemental_table_3_suppst3.pdf]

Supplemental Table 3. Normalization Subset of Cohort 1. Description of the 50 common samples analyzed across Cohort 1 and Cohort 2 to enable normalization. The 50 samples were used in Cohort 1 for feature selection and modeling, but in Cohort 2, they were used only for normalization, not for modeling.

Diagnosis	Group	Count
Cancer	All OC	26
	Early-Stage OC	9
	Late-Stage OC	17
Non-Cancer	All Controls	24
	Normal	8
	Benign Conditions	16
**Grand Total:**		**50**
